# Resolving content moderation dilemmas between free speech and harmful misinformation

**DOI:** 10.1073/pnas.2210666120

**Published:** 2023-02-07

**Authors:** Anastasia Kozyreva, Stefan M. Herzog, Stephan Lewandowsky, Ralph Hertwig, Philipp Lorenz-Spreen, Mark Leiser, Jason Reifler

**Affiliations:** ^a^Center for Adaptive Rationality, Max Planck Institute for Human Development, Berlin 14195, Germany; ^b^School of Psychological Science, University of Bristol, Bristol BS8 1QU, United Kingdom; ^c^School of Psychological Sciences, University of Western Australia, Perth 6009, Australia; ^d^Amsterdam Law and Technology Institute, VU-Amsterdam, Amsterdam 1081 HV, The Netherlands; ^e^Department of Politics, University of Exeter, Exeter EX4 4PY, United Kingdom

**Keywords:** moral dilemma, harmful content, online speech, content moderation, conjoint experiment

## Abstract

In online content moderation, two key values may come into conflict: protecting freedom of expression and preventing harm. Robust rules based in part on how citizens think about these moral dilemmas are necessary to deal with this conflict in a principled way, yet little is known about people’s judgments and preferences around content moderation. We examined such moral dilemmas in a conjoint survey experiment where US respondents (*N* = 2, 564) indicated whether they would remove problematic social media posts on election denial, antivaccination, Holocaust denial, and climate change denial and whether they would take punitive action against the accounts. Respondents were shown key information about the user and their post as well as the consequences of the misinformation. The majority preferred quashing harmful misinformation over protecting free speech. Respondents were more reluctant to suspend accounts than to remove posts and more likely to do either if the harmful consequences of the misinformation were severe or if sharing it was a repeated offense. Features related to the account itself (the person behind the account, their partisanship, and number of followers) had little to no effect on respondents’ decisions. Content moderation of harmful misinformation was a partisan issue: Across all four scenarios, Republicans were consistently less willing than Democrats or independents to remove posts or penalize the accounts that posted them. Our results can inform the design of transparent rules for content moderation of harmful misinformation.

## Significance

Content moderation of online speech is a moral minefield, especially when two key values come into conflict: upholding freedom of expression and preventing harm caused by misinformation. Currently, these decisions are made without any knowledge of how people would approach them. In our study, we systematically varied factors that could influence moral judgments and found that despite significant differences along political lines, most US citizens preferred quashing harmful misinformation over protecting free speech. Furthermore, people were more likely to remove posts and suspend accounts if the consequences of the misinformation were severe or if it was a repeated offense. Our results can inform the design of transparent, consistent rules for content moderation that the general public accepts as legitimate.

We have a right to speak freely. We also have a right to life. When malicious disinformation—claims that are known to be both false and dangerous—can spread without restraint, these two values collide head-on.—George Monbiot (1).

[W]e make a lot of decisions that affect people’s ability to speak. [...] Frankly, I don’t think we should be making so many important decisions about speech on our own either.—Mark Zuckerberg (2).

Every day, human moderators and automated tools make countless decisions about what social media posts can be shown to users and what gets taken down, as well as how to discipline offending accounts. The ability to make these content moderation decisions at scale, thereby controlling online speech, is unprecedented in human history. Legal requirements make some content removal decisions easy for platforms (e.g., selling illegal drugs or promoting terrorism). But what about when content is not explicitly illegal but rather “legal but harmful” or “lawful but awful”? Harmful misinformation—inaccurate claims that can cause harm—falls into this category. False and misleading information is considered harmful when it undermines people’s ability to make informed choices and when it leads to adverse consequences such as threats to public health or the legitimacy of an election (3).

The scale and urgency of the problems around content moderation became particularly apparent when Donald Trump and political allies spread false information attacking the legitimacy of the 2020 presidential election, culminating in a violent attack on the US Capitol. Subsequently, most major social media platforms suspended Trump’s accounts (4–6). After a sustained period of prioritizing free speech and avoiding the role of “arbiters of truth” (2, 7), social media platforms appear to be rethinking their approach to governing online speech (8). In 2020, Meta overturned its policy of allowing Holocaust denial and removed some white supremacists groups from Facebook (9); Twitter implemented a similar policy soon after (10). During the COVID-19 pandemic, most global social media platforms took an unusually interventionist approach to false information and vowed to remove or limit COVID-19 misinformation and conspiracies (11–14)—an approach which might undergo another shift soon (see ref. 15). In October 2021, Google announced a policy forbidding advertising content on its platforms that “mak[es] claims that are demonstrably false and could significantly undermine participation or trust in an electoral or democratic process” or that “contradict[s] authoritative, scientific consensus on climate change” (16). And most recently, Pinterest introduced a new policy against false or misleading climate change information across both content and ads (17). (An overview of major platforms’ moderation policies related to misinformation is provided in *SI Appendix*, Table S9.)

At the core of these decisions is a moral dilemma: Should freedom of expression be upheld even at the expense of allowing dangerous misinformation to spread, or should misinformation be removed or penalized, thereby limiting free speech? When choosing between action (e.g., removing a post) and inaction (e.g., allowing a post to remain online), decision-makers face a choice between two values (e.g., public health vs. freedom of expression) that, while not in themselves mutually exclusive, cannot be honored simultaneously. These cases are moral dilemmas: “situations where an agent morally ought to adopt each of two alternatives but cannot adopt both” (18, p. 5).

Although moral dilemmas have long been used in empirical studies of ethics and moral decision-making, moral dilemmas in online content moderation are relatively new. Yet insights into public preferences are necessary to inform the design of consistent content moderation policies and grant legitimacy to policy decisions. Here, we begin to bridge this gap by studying public preferences around content moderation and investigating what attributes of content moderation dilemmas impact people’s decisions the most.

Resolving content moderation dilemmas is difficult. Mitigating harms from misinformation by removing content and deplatforming accounts (especially at scale) might challenge the fundamental human right to “receive and impart information and ideas through any media and regardless of frontiers” (19, art. 19). Moreover, there are good reasons why existing legal systems protect even false speech (20). People with the power to regulate speech based on its accuracy may succumb to the temptation to suppress opposition voices (e.g., authoritarian rulers often censor dissent by determining what is “true”). Censoring falsehoods might also prevent people from freely sharing their opinions, thereby deterring (e.g., due to fear of punishment) even legally protected speech (21). Indeed, a core tenet of the marketplace of ideas is that it can appropriately discard false and inaccurate claims: “The best test of truth is the power of an idea to get itself accepted in the competition of the market” (22).

Do digital and social media, where harmful misinformation can quickly proliferate and where information flow is algorithmically moderated, belie this confidence in the marketplace of ideas? As Sunstein (20) argued, “far from being the best test of truth, the marketplace ensures that many people accept falsehoods” (p. 49). For instance, when a guest on Joe Rogan’s popular podcast shared discredited claims about COVID-19 vaccines, he spread potentially fatal misinformation to millions of listeners (23). Here, two important points must be distinguished: First, while some types of misinformation may be relatively benign, others are harmful to people and the planet. For example, relative to factual information, in the United Kingdom and the United States, exposure to misinformation can reduce people’s intention to get vaccinated against COVID-19 by more than 6% points (24). This fact may justify invoking Mill’s principle of harm (25, 26), which can be invoked to warrant limiting freedom of expression in order to prevent direct and imminent harm to others. Second, sharing one’s private opinions, however unfounded, with a friend is substantially different from deliberately sharing potentially harmful falsehoods with virtually unlimited audiences. One may therefore argue that freedom of speech does not entail “freedom of reach” (27) and that the right to express one’s opinions is subject to limitations when the speech in question is amplified online.

Freedom of expression is an important right, and restrictions on false speech in liberal democracies are few and far between. State censorship is a trademark of authoritarianism: The Chinese government’s censorship of Internet content is a case in point (28), as is the introduction of “fake news” laws during the pandemic as a way for authoritarian states to justify repressive policies that stifle the opposition and further infringe on freedom of the press (29–31) (for an overview of misinformation actions worldwide, see ref. 32). Furthermore, in March 2022, the Russian parliament approved jail terms of up to 15 y for sharing “fake” (i.e., contradicting the official government position) information about the war against Ukraine, which led many foreign and local journalists and news organizations to limit coverage of the invasion or withdraw from the country entirely.

Unlike in authoritarian or autocratic countries, in liberal democracies, online platforms themselves are the primary regulators of online speech. This responsibility raises the problem of rule-making powers being concentrated in the hands of a few unelected individuals at profit-driven companies. Furthermore, platforms increasingly rely on automated content moderation; for instance, the majority of hate speech on Facebook is removed by machine-learning algorithms (33). Algorithmic content moderation at scale (34) poses additional challenges to an already complicated issue, including the inevitable occurrence of false positives (when acceptable content is removed) and false negatives (when posts violate platform policies but escape deletion). Algorithms operate on the basis of explicit and implicit rules (e.g., should they remove false information about climate change or only about COVID-19?). Content moderation—either purely algorithmic or with humans in the loop—inevitably requires a systemic balancing of individual speech rights against other societal interests and values (8).

Scenarios involving moral dilemmas (e.g., the trolley problem) are used widely in moral psychology to assess people’s moral intuitions and reasoning (35), and experiments featuring moral dilemmas are an established approach to studying people’s moral intuitions around algorithmic decision-making (36, 37) and computational ethics (38). Classical dilemmas include scenarios involving choices between two obligations arising from the same moral requirement or from two different moral requirements. Most studies focus on dilemmas of the sacrificial type: Presenting a choice within one moral requirement (e.g., saving lives) with asymmetrical outcomes (e.g., to save five lives by sacrificing one; see refs. 39 and 40). Content moderation decisions, however, represent a different, and largely unstudied, problem: dilemmas between two different values or moral requirements (e.g., protecting freedom of expression vs. mitigating potential threats to public health) that are incommensurate and whose adverse outcomes are difficult to measure or quantify.

We constructed four types of hypothetical scenarios arising from four contemporary topics that are hotbeds of misinformation: politics (election denial scenario), health (antivaccination scenario), history (Holocaust denial scenario), and the environment (climate change denial scenario). In designing these scenarios, we relied on the current content moderation policies of major social media platforms and selected topics where active policies on misinformation have already been implemented (*SI Appendix*, Table S9).

**Fig. 1.**
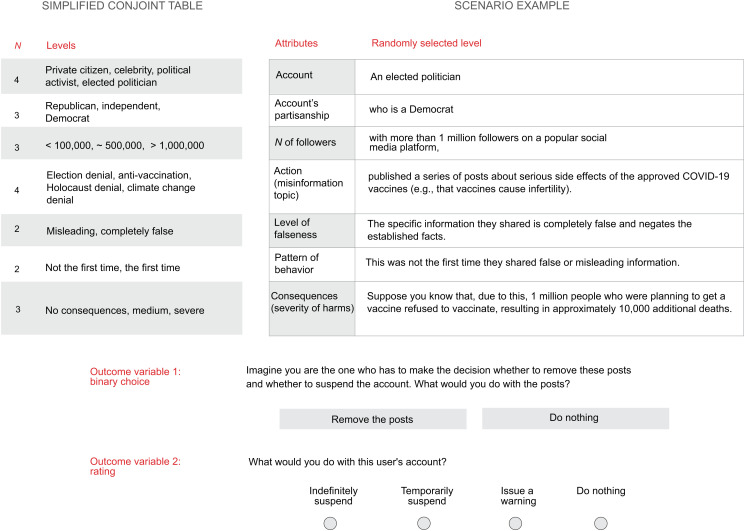
Complete listing of all attribute levels in *SI Appendix*, Table S2.

We used a single-profile conjoint survey experiment to explore what factors influence people’s willingness to remove false and misleading content on social media and to penalize accounts that spread it. A conjoint design is particularly suitable for such a multilevel problem, where a variety of factors can impact decision-making (41, 42). Factors we focused on are characteristics of the account (the person behind it, their partisanship, and the number of followers they have), characteristics of the shared content (the misinformation topic and whether the misinformation was completely false or only misleading), whether this was a repeated offense (i.e., a proxy for intent), and the consequences of sharing the misinformation. All these factors were represented as attributes with distinct levels (Fig. 1). This design yielded 1,728 possible unique cases.

In the conjoint task, each respondent (*N* = 2,564) faced four random variations of each of the four scenario types (Fig. 1 for an example), thus deciding on 16 cases altogether (40,845 evaluations in total, after missing responses were removed). Each scenario type represented a different misinformation topic (election denial for politics, antivaccination for health, Holocaust denial for history, and climate change denial for environment), with consequences adjusted for each topic. For each case, respondents were asked to make two choices: whether to remove the posts mentioned in the scenario and whether to suspend the account that posted them. We recruited 2,564 US respondents via the Ipsos sample provider between October 18 and December 3, 2021. The sample was quota-matched to the US general population. The full experimental design and sample information are described in the *Materials and Methods* section.

## Results

### A. Restricting Misinformation: Decisions to Remove Posts and Penalize Accounts.

**Fig. 2.**
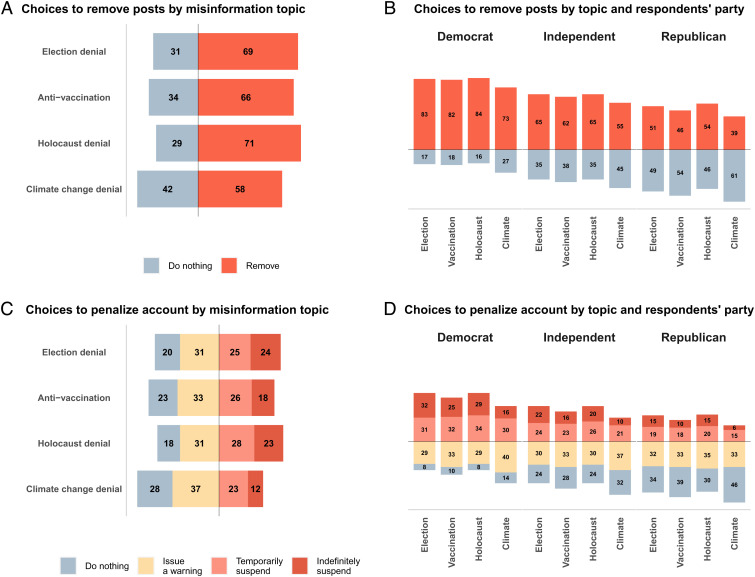
Proportion of choices to remove posts and to suspend accounts. All numeric values represent percentages. (*A*) Choices to remove posts or do nothing by misinformation topic (all cases). (*B*) Choices to remove posts or do nothing, by topic and respondents’ party affiliation. (*C*) Choices to penalize account by misinformation topic (all cases). (*D*) Choices to penalize account by topic and respondents’ party affiliation. N= 40,845 cases evaluated in total. (Cases evaluated by Democrats, including Democrat-leaning, n= 19,338; by independents n= 8,229; by Republicans, including Republican-leaning, n= 13,278). For confidence intervals and proportions to suspend account (dichotomized rating: do nothing/issue a warning vs. temporarily/indefinitely suspend), *SI Appendix*, Fig. S1.

For the majority of cases, across all four topics, most respondents chose to remove posts featuring false or misleading information (Fig. 2*A*). Climate change denial was removed the least (58%), whereas Holocaust denial was removed the most (71%), closely followed by election denial (69%) and antivaccination content (66%). In deciding whether to do nothing, issue a warning, temporarily suspend the account or indefinitely suspend it, the majority of respondents preferred to issue a warning (between 31% and 37% across all four topics; Fig. 2*C*). However, the total number of choices to temporarily or indefinitely suspend an account constituted about half of responses in the Holocaust denial (51%) and election denial (49%) scenarios, followed by antivaccination (44%) and climate change denial scenarios (35%; *SI Appendix*, Fig. S1). Thus, even though respondents prioritized taking an action, they were on average less likely to suspend accounts than to remove posts.

Fig. 2 *B* and *D* shows a clear difference between Democrats and Republicans, with independents in between. Only a small minority of Democrats chose to leave misinformation in place or to not take action against the account spreading it. Republicans were almost evenly split in their decisions to remove the posts in three of the four scenarios; in the climate change denial scenario, a majority of Republican respondents preferred to do nothing. The majority of Republicans and independents chose to do nothing or to issue a warning rather than penalize the account (*SI Appendix*, Fig. S1).

### B. Conjoint Analyses: What Influences Content Moderation Decisions?.

**Fig. 3.**
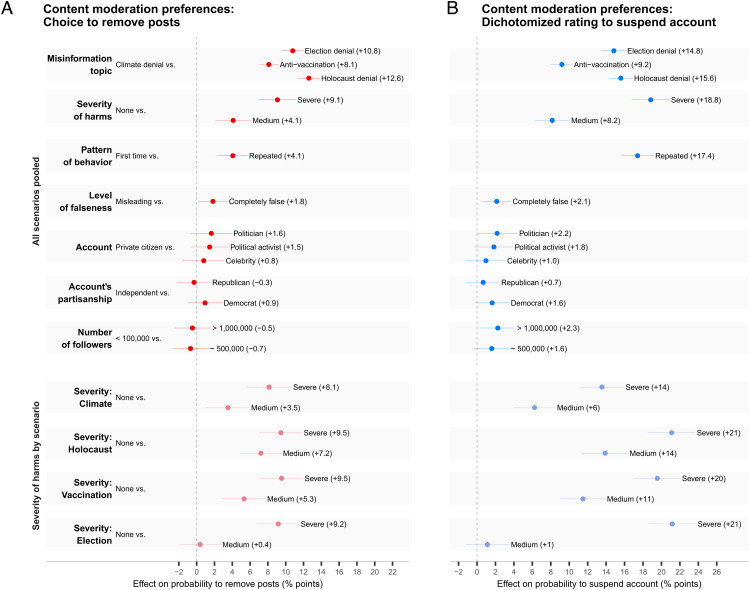
Preferences for content moderation. The figure reports average marginal component effects (AMCEs) plotted with 95% confidence intervals. In each row, effect sizes show an impact of each attribute level (*Right*) relative to the reference attribute level (*Left*), aggregated over all other attributes. AMCEs are converted to percentage points and represent effects on the probability to remove the posts (*A*) and on the probability to suspend the account (*B*). In (*B*) the 4-point rating outcome variable is dichotomized (do nothing or issue a warning [0] vs. temporarily or indefinitely suspend an account [1]). In both *A* and *B*, “all scenarios pooled” displays all attributes, including severity of harms, in a pooled manner. As, in each scenario, the consequences were matched to the respective misinformation topic (and thus, unlike all other attributes, were not common across topics), “severity of harms by scenario” shows scenario-specific effects for this attribute. *SI Appendix*, Table S2 for the topic-matched, verbatim phrasings of the levels for this attribute. For all AMCE and marginal means estimates, *SI Appendix*, Tables S3–S8.

To analyze respondents’ content moderation preferences related to different conjoint factors, we computed average marginal component effects (AMCEs) for both outcome variables: the binary choice to remove the posts and the rating of how to handle the accounts (dichotomized to a binary decision: do nothing/issue a warning or temporarily/indefinitely suspend). Fig. 3 shows pooled results across all scenarios (i.e., the four scenario types are treated as the levels of the “misinformation topic” attribute; *SI Appendix*, Table S2). For the attribute “Severity of harm,” scenario-level results are also shown. Results for the nondichotomized rating variable are displayed in *SI Appendix*, Fig. S2; full scenario-level results are reported in *SI Appendix*, Figs. S3–S5.

Three attributes had the largest effects on people’s content removal decisions: misinformation topic, severity of harm, and pattern of behavior. The misinformation topic consistently produced the largest effect for the choice to remove posts. As Fig. 3 shows, changing the misinformation topic from climate change denial to Holocaust denial increased the probability of removing the posts by 13% points and the probability of suspending the account by 16% points.

The second-strongest effect was produced by severity of harm: The more harmful the consequences of sharing misinformation (e.g., lives lost), the more likely respondents were to act. For instance, changing the severity of consequences from none to severe across scenarios increased the probability of choosing to remove the posts by 9% points (Fig. 3*A*). The effect of severity of harm was strongest for the dichotomized variable to suspend accounts: Changing the severity of consequences from none to severe across scenarios increased the probability to suspend the account by 19% points (Fig. 3*B*).

Note that the “severity of harm” attribute, which represents the consequences of spreading misinformation, was matched to each topic and thereby differed across topics. In the election denial scenario, the severe consequences were “a violent demonstration occurred, five people died, and 150 protesters were detained,” whereas medium-level consequences were “a nonviolent demonstration occurred.” In the Holocaust denial scenario, the medium level was “several anti-Semitic attacks occurred, with no severe injuries,” and the severe level was “several anti-Semitic attacks occurred, injuring two people and killing one person.” (For all phrasings, *SI Appendix*, Table S2.) Although consequences across topics were noncommensurate (e.g., in terms of number of casualties), they followed the same pattern of increasing severity (none, medium, severe)—this was also reflected in respondents’ posttreatment ratings of the outcomes’ severity (*SI Appendix*, Fig. S12).

The lower section of Fig. 3 shows how the effects of severity of harm varied across the four scenarios. Here, the severe levels for Holocaust denial, election denial, and antivaccination produced the largest effects. In the election denial scenario, changing the severity of harm from “none” to “medium” (peaceful demonstration) had no effect on the decision to remove posts and suspend accounts, while in the other three scenarios, the medium level of consequences led to significantly higher rates of removal and suspension relative to no consequences. This pattern across scenarios was also reflected in the posttreatment subjective rating of outcome severity, where the majority of participants rated a nonviolent demonstration (medium level) as not at all or only slightly severe (69%) and a violent demonstration with casualties (severe level) as extremely or very severe (74%; *SI Appendix*, Fig. S12).

A third important factor affecting content moderation preferences was the pattern of behavior. Changing this attribute from first offense to repeated offense increased the probability of removing the posts by 4 percentage points and increased the probability of suspending the account by 17 percentage points.

In sum, for decisions about both posts and accounts, the topic of the misinformation, the severity of the outcomes, and whether it was a repeated offense had the strongest impact on decisions to remove posts and suspend accounts. Attributes related to an account’s features—the person behind it, their partisanship, and the number of followers—and whether the information was misleading or completely false had relatively little impact on respondents’ decisions.

### C. Subgroup Analyses by Partisanship and Attitudes Toward Free Speech.

We conducted subgroup analyses for two main characteristics of interest, respondents’ political partisanship and their attitude toward freedom of expression, in order to assess how they affected respondents’ content moderation preferences.

**Fig. 4.**
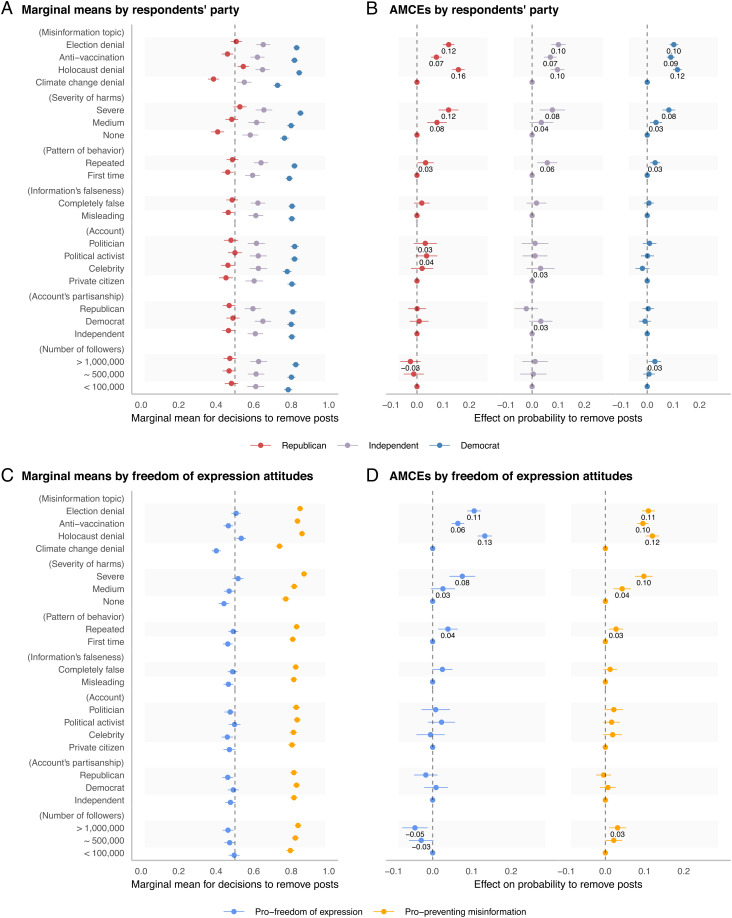
Respondent subgroup analyses: Differences by political identification and attitudes toward freedom of expression. For three partisan subgroups, (*A*) marginal means point estimates represent the average likelihood of decisions to remove the posts for each attribute level, and (*B*) average marginal component effects (AMCEs) represent effects on the probability to remove the posts. For two freedom of expression subgroups, (*C*) marginal means point estimates represent the average likelihood of decisions to remove the posts for each attribute level, and (*D*) AMCEs represent effects on the probability to remove the posts. Dashed lines represent the null effect; marginal means point estimates and AMCEs plotted with 95% confidence intervals.

Fig. 4 *A* and *B* shows marginal means and AMCEs for the choice to remove the posts for three subgroups: Republicans (including Republican-leaning respondents), independents, and Democrats (including Democrat-leaning respondents; *SI Appendix*, Table S1 for their distribution in the sample). Subgroup results by partisanship for penalizing accounts, both continuous and dichotomized, are shown in *SI Appendix*, Figs. S6 and S7.

Marginal means in Fig. 4*A* show that the three partisan subgroups had different content moderation preferences. Republicans were least likely to remove posts for all attribute levels, whereas Democrats and independents were more likely to remove the posts than to do nothing. The only attribute level that made Republicans more likely to remove the posts rather than leave them up was Holocaust denial.

The AMCEs in Fig. 4*B* show how attribute levels affected the probability of removing posts by respondents’ party identification. All three groups showed similar patterns, with two exceptions. First, a large number of followers (i.e., > 1,000,000 relative to the reference level of < 100,000) affected the judgments of Democrats and independents, but not those of Republicans. For Democrats, a larger reach increased the probability of removing the posts by 3 percentage points (Fig. 4*B*) and the probability of suspending the account by 6 percentage points (*SI Appendix*, Fig. S7). For independents, a larger reach did not increase the probability of removing the posts (mirroring the results for Republicans), but it did increase the probability of suspending the account by 5 percentage points (mirroring the results for Democrats; *SI Appendix*, Fig. S7). Second, in decisions to penalize accounts, Democrats penalized repeat-offender accounts more strongly than Republicans and independents (*SI Appendix*, Figs. S6 and S7). Finally, contrary to our expectations, there was no clear indication of a partisanship effect. Participants were not more inclined to remove posts from an account that was at odds with their own political leaning, nor were they more likely to suspend accounts that did not match their political preferences. There was one exception, however: Independents were more likely to suspend accounts from Democrats (*SI Appendix*, Fig. S7), although they were not more likely to remove posts from Democrats.

Fig. 4 *C* and *D* shows marginal means and AMCEs for the choice to remove the posts for two subgroups: pro-freedom of expression and pro-mitigating harmful misinformation. These subgroups were formed based on responses to our pretreatment question: “If you absolutely have to choose between protecting freedom of expression and preventing disinformation from spreading, which is more important to you?” A total of 47% of respondents indicated that freedom of expression was more important. More Republicans were pro-freedom of expression (64%) and more Democrats were pro-mitigating harmful misinformation (66%; *SI Appendix*, Fig. S10 for details, and *SI Appendix*, Figs. S8 and S9 for subgroup results for the ratings to penalize accounts, continuous and dichotomized).

Marginal means in Fig. 4*C* show that participants made decisions that were consistent with their attitudes. On average, those who favored freedom of expression were equally or less likely to remove posts than they were to do nothing, whereas those who favored mitigation of misinformation were much more likely to remove posts than they were to do nothing. For decisions to penalize accounts (which overall was, on average, less popular than removing posts), respondents who were pro-mitigating misinformation were more likely than not to suspend accounts, except for three attribute levels: First offense, no harmful consequences, and climate change denial (*SI Appendix*, Fig. S9).

The AMCEs in Fig. 4*D* show how attribute levels affected the probability of removing posts by respondents’ attitudes toward freedom of expression. Notably, respondents who valued freedom of expression over mitigating harmful misinformation were less likely to remove posts by accounts with many followers, whereas respondents who indicated that preventing misinformation was more important than protecting free speech were more likely to remove posts by accounts with many followers. This pattern was only partly preserved in the decision to penalize accounts; namely, pro-mitigating respondents had a higher likelihood to penalize accounts with a bigger reach (*SI Appendix*, Figs. S8 and S9).

### D. Role of Partisanship and Accuracy of Beliefs.

Toward the end of the survey, we assessed respondents’ beliefs regarding a variety of claims relevant to the scenarios in order to examine the role of accuracy of their existing knowledge. Republicans were more likely than Democrats or independents to believe inaccurate claims and disbelieve accurate claims (*SI Appendix*, Fig. S10). For instance, 75% of Democrats rated the inaccurate statement “The FDA-approved COVID-19 vaccines can cause infertility” as definitely or possibly false, versus 50% of Republicans. The most polarizing inaccurate statement—“The 2020 US presidential election was stolen from Donald Trump”—was rejected by 84% of Democrats but only by 32% of Republicans. Similarly, the accurate statement “There is an overwhelming scientific consensus that human activity (e.g., burning fossil fuels) is the leading cause of climate change” was endorsed by 78% of Democrats but only by 36% of Republicans. The only notable exception was the statement related to Holocaust denial, where—irrespective of partisanship—only about 5% of respondents rejected the accurate claim “It is a well established historical fact that 6 million Jews died in the Holocaust” as definitely or probably false (*SI Appendix*, Fig. S10). Since this specific topic did not produce differences along partisan lines, it is of particular interest to our analyses. As Fig. 4 shows, Holocaust denial was the only topic in which a majority of respondents in each partisan group, as well as the majority of pro-free speech respondents, decided to remove posts. In our choice of topics for the conjoint design, we aimed to increase external validity by focusing on issues relevant to political discourse and content moderation at the time of our study, which has the consequence that respondents may have had elite cues available affecting their responses. The partisan difference results we observe could vary as topics and issues (and the availability of elite cues) vary.

The finding that Republicans were more likely to endorse inaccurate claims relevant to our scenarios raises an important question: To what extent do partisan differences in content moderation reflect genuine differences in how respondents weighed opposing objectives and values in the moral dilemmas and to what extent do they merely reflect different beliefs about the accuracy of the shared content? Assuming that all respondents, irrespective of partisanship, are less likely to remove posts or suspend accounts if they deem the posted content to be truthful, this alone would predict that Republicans would intervene less.

To test the plausibility of this alternative explanation for the partisan differences that emerged, we conducted robustness analyses, subsetting responses in the conjoint part of our study by respondents’ beliefs in the corresponding misinformation statements. This allowed us to compare content moderation preferences between two groups: respondents with accurate beliefs and respondents with inaccurate or uncertain beliefs (*SI Appendix*, *Appendix C* for details). In this subset of responses (*SI Appendix*, Fig. S17), a large gap emerged: Many more respondents with accurate beliefs than respondents with inaccurate or uncertain beliefs opted to remove false and misleading posts and penalize the accounts that spread them. This difference, however, was much smaller for the Holocaust denial scenario. The majority of Republicans with accurate beliefs were also more likely than Republicans with inaccurate or uncertain beliefs to take action against online misinformation. Importantly, however, the main patterns in the subgroup differences remained robust, including the finding that Republicans were less likely than independents and Democrats to take action against misinformation.

It is important to keep in mind that because these analyses are correlational—that is, the accuracy of beliefs is an endogenous (i.e., nonrandomized) variable—they do not license causal claims about the effects of the accuracy of respondents’ beliefs on their content moderation decisions. Rather, the purpose of these analyses is to challenge our findings on partisanship and content moderation. To go beyond correlational analyses and explore potential causal effects of misinformation beliefs and partisanship, we conducted a set of moderation analyses (based on ref. 43). This approach allowed us to estimate causal moderation effects of a nonrandomized independent variable (e.g., accuracy of beliefs or respondent partisanship) on a dependent variable (e.g., decisions to remove posts and suspend accounts) for randomized conjoint attributes. Details of these analyses for choice to remove posts (*SI Appendix*, Fig. S18) and to suspend accounts (*SI Appendix*, Fig. S19) are presented in *SI Appendix*, *Appendix C*.

We found no clear pattern in the moderation effects, and only few moderation effects were statistically significant. For instance, for decisions to suspend accounts in the attributes “severity of harm,” “pattern of behavior,” and “number of followers,” respondents who endorsed inaccurate beliefs relevant to the topic at hand were, on average, less sensitive to changes in the severity of consequences, in whether it was a repeated or first offense, and the number of followers of the account (*SI Appendix*, Fig. S19*A*). However, moderation effects of partisanship pointed in the same direction only for the “pattern of behavior” and “number of followers” attributes in decisions to suspend accounts. In decisions to remove posts, they followed, if anything, the opposite pattern to that found for the “severity of harm” attribute—namely, Republicans were more sensitive than Democrats to the “severity of harm” attribute when consequences of sharing misinformation were severe (relative to none; *SI Appendix*, Fig. S18*B*).

Taken together, both robustness checks in the subset analyses and causal moderation analyses show that misinformation beliefs play a role in content moderation decisions but do not support the claim that these beliefs offer a viable explanation for the substantial differences in content moderation preferences between Republicans and Democrats that we observed.

## Discussion

Content moderation is controversial and consequential. Regulators are reluctant to restrict harmful but legal content such as misinformation, thereby leaving platforms to decide what content to allow and what to ban. At the heart of policy approaches to online content moderation are trade-offs between fundamental values such as freedom of expression and the protection of public health. In our investigation of which aspects of content moderation dilemmas affect people’s choices about these trade-offs and what impact individual attitudes have on these decisions, we found that respondents’ willingness to remove posts or to suspend an account increased with the severity of the consequences of misinformation and whether the account had previously posted misinformation. The topic of the misinformation also mattered—climate change denial was acted on the least, whereas Holocaust denial and election denial were acted on more often, closely followed by antivaccination content. In contrast, features of the account itself—the person behind the account, their partisanship, and number of followers—had little to no effect on respondents’ decisions. In sum, the individual characteristics of those who spread misinformation mattered little, whereas the amount of harm, repeated offenses, and type of content mattered the most.

Generally speaking, these results provide support for a consequentialist approach to content moderation of online misinformation. Although we did not measure participants’ moral attitudes, their preferences are compatible with a consequentialist approach. Consequentialism judges the moral permissibility of actions based on their outcomes (44). In utilitarianism, a paradigmatic version of moral consequentialism, maximizing happiness (45) and minimizing harms for most people (46) are key ethical principles. Notably, minimizing harm is one of the most universal ethical principles (e.g., ref. 47). The results of our study support the idea that it holds for online content moderation of misinformation as well; minimizing harm has also been found to be important for the moderation of hate speech (48). An internal survey by Twitter in 2019 showed that people support penalties for harmful content online: More than 90% of an international sample supported removing misleading and altered content when it was clearly intended to cause certain types of harm, and more than 75% believed that accounts sharing false and misleading information should be punished, for instance by deleting their Tweets or suspending the account (49).

Repeated offense can be classified as character evidence—evidence that suggests that a person is likely or unlikely to have acted a certain way based on their reputation, prior conduct, or criminal history. According to our data, repeated sharing of misinformation is a crucial factor in people’s decisions to remove posts and penalize accounts. Repeated offenses can signal malicious intent, which in turn lends support to the idea that people tend to penalize misinformation shared with malicious intent more than misinformation that might have been shared unwittingly. It is also possible that people are inclined to punish repeated sharing of falsehoods because they consider the potential amplification of harm brought about by repeated sharing (e.g., due to increased exposure to false claims).

Another relevant feature was the number of followers an account had, which is a strong determinant of its reach. Although this feature mattered little on the aggregate level, it was important in varying ways to different subgroups: Respondents who were pro-freedom of expression were less likely to penalize accounts with many followers. When prioritizing freedom of expression over the mitigation of harmful misinformation, it seems that accounts with more reach (over 1,000,000 followers in our scenarios) are thought to deserve more protection. In contrast, Democrats and respondents who were pro-mitigating misinformation were more likely to penalize accounts with many followers. Even though these decisions are contradictory, they are coherent in light of respondents’ professed values.

Partisan differences played a major role in people’s decisions on content moderation. Respondents did not penalize political out-group accounts more than in-group accounts, but Republicans and Democrats did, in general, make different trade-offs to resolve the dilemma between protecting free speech and removing potentially harmful misinformation. Democrats showed a stronger preference for preventing dangerous falsehoods across all four scenarios, whereas Republicans preferred to protect free speech and imposed fewer restrictions. This partisan divide is consistent with other surveys showing stark partisan divisions in attitudes toward the role of governments and tech firms in restricting online misinformation (50).

Given the extent of political polarization in the United States (see, e.g., ref. 51), it would have been surprising if Democrats, Republicans, and independents had uniformly supported the same content moderation measures. And yet, in the majority of cases across the four scenarios, respondents in our study chose to remove the posts. Respondents were less willing to suspend offending accounts but nevertheless preferred taking some action to doing nothing. For instance, in the election denial scenario, 49% of respondents chose to temporarily or indefinitely suspend the account, and 31% chose to issue a warning. Assuming that an unheeded warning will eventually be followed by temporary or indefinite suspension, this response pattern implies that even in this highly contentious issue, 80% of respondents prefer taking action over doing nothing. Moreover, causal effects of the levels in most attributes were comparable across partisan groups.

Partisan differences in attitudes toward freedom of expression could be rooted in differing approaches to choice autonomy. Republicans’ views may be rooted in libertarian philosophy, where individual rights and autonomy are paramount. Democrats’ views, however, may be rooted in a modern liberalism that prioritizes social justice such that individual rights can be limited for the benefit of society as a whole (52). These differences in political philosophy might help account for differences between Republicans’ and Democrats’ attitudes toward removing potentially harmful misinformation online.

Another factor that might account for partisan differences relates to differences in beliefs about the facts at hand. Our study revealed significant partisan divides in respondents’ beliefs. Only in the Holocaust denial scenario did beliefs converge across all three partisan subgroups. Notwithstanding existing differences in beliefs, our robustness checks showed that partisan differences remained even when considering only respondents with accurate beliefs about the relevant background knowledge in a scenario (e.g., who correctly dismissed a claim such as “The FDA-approved COVID-19 vaccines can cause infertility”). Causal moderation analyses did not provide any evidence that partisan differences in beliefs about the facts are a viable alternative explanation for the observed partisan differences.

Our study had some limitations. First, in three of four scenarios, Republicans disagree about the ground truth more than Democrats. This is a side effect of including topics that are addressed in the content moderation policies of major platforms (for an overview, *SI Appendix*, *Appendix D*). Although a range of conspiracy beliefs are endorsed by both Democrats and Republicans, for example, that genetically modified organisms are dangerous; that the measles, mumps, and rubella vaccination causes autism; and that the Holocaust never happened (53), only Holocaust denial and antivaccination—both surveyed in our study—are explicitly covered in platforms’ regulations. Considering a wider range of topics, with different patterns of agreement across the political spectrum (53), merits further exploration. Second, because both our scenarios and our respondents were based in the United States, the generalizability of our conclusions is restricted. We chose to focus on the US context for two reasons. One is that free speech protectionism is a distinct feature of American culture and politics, and Americans are more supportive of all forms of freedom of expression than are citizens of other countries (54). The other reason is that the current debate around content moderation is mostly centered in the United States, and many of the rules are being established by US-based companies. However, irrespective of who makes the rules, content moderation affects people across countries and cultures. Ideally, future studies will cover a broader range of cultures and countries. Third, in our conjoint experiment, we stipulated that a user’s actions would lead to a specific consequence. In real life, however, the consequences of a social media post are much harder to establish. Future research should address the role of this uncertainty. Finally, we focused on only one type of content moderation dilemmas: when removing harmful but legal content compromises the right to free speech or, conversely, protecting free speech comes at the cost of social harm. But there are many others; for instance, policing illegal content (e.g., specific types of pornographic content) through social media raises a dilemma between public safety and individual privacy (55).

When considering the implications of our results for policy, it is important to keep in mind that in liberal democracies, policymakers are reluctant to regulate legal but not harmful misinformation at the risk of limiting freedom of expression (3, 56). The principle of proportionality requires that harsh measures should be applied only when strictly necessary and that a variety of less intrusive mitigating tools should be implemented as a first line of defense. For example, instead of immediately removing harmful misinformation, a range of less intrusive measures can be introduced, including warning labels, fact-checking labels, and other prompts that slow the spread of falsehoods (57, 58). However, content moderation of harmful content is a standard practice and one that should be improved: Platforms require a common policy that is developed and implemented transparently and consistently.

Our findings present both opportunities and challenges for policy-making. The fact that respondents did not focus on the characteristics of the accounts, but rather on the factors related to the offense itself, is arguably consistent with nonpartiality and nondiscrimination. The small impact of the level of falseness of a post also indicates that the public is less sensitive to the subtle differences between information being factually false or merely distorted and much more focused on the amount of harm that can follow from sharing misinformation and whether it was a repeated offense. Severity of harm and repeated offense were especially important factors in decisions to suspend accounts, where, in general, respondents were more reluctant to intervene than they were in decisions regarding posts. This suggests that suspending accounts requires more care in implementation and a higher standard for proving offense. However, this approach is largely consistent with existing policies; for instance, several platforms, including Meta, have policies for repeat offenders (59). In contrast, our finding that people’s tendency to intervene varies widely across misinformation topics (e.g., climate change denial vs. Holocaust denial) points to a potential hurdle for policymakers in that they are faced with the challenge of determining how harmful misinformation is in different domains by assessing the potential consequences.

Online platforms, as gatekeepers of online content (60), cannot simply assume that their users will endorse their moderation policies. Instead, according to the theory of network communitarianism (61), effective policy-making requires recognizing the Internet community as necessary to legitimizing any approach to content moderation. Protecting the public from the harms of misinformation requires that policymakers recognize the role of the active community and map the actions that the community would take when faced with similar content moderation decisions. Measuring and understanding the public’s preferences around content moderation would thus help establish a relevant, evidence-based starting point for a conversation between policymakers and the public. Furthermore, a systematic assessment of public opinion on content moderation, as our study offers, leaves less rhetorical wiggle room for individuals with vested interests (e.g., politicians, CEOs) to make and get away with self-serving claims about what users or the public wants. People’s preferences are not the only benchmark for making important trade-offs on content moderation, but ignoring their preferences altogether risks undermining the public’s trust in content moderation policies and regulations. Results such as those presented here can contribute to the process of establishing transparent and consistent rules for content moderation that are generally accepted by the public.

## Materials and Methods

### Sample.

An online survey of US participants (*N*= 2,564) was fielded by Ipsos Observer between October 18 and December 3, 2021. The panel provider uses multisource recruitment and compensates their panelists with points, which are redeemable for a variety of rewards. The sample was quota-matched to the US general population in terms of age, gender, education, ethnicity, and region of residence, with two exceptions where it proved to be infeasible to fill quotas in the online sample: Hispanics (ethnicity quota) and people without a high school education (education quota). *SI Appendix*, Table S1 for the demographic distribution of the sample and information on how it compares to the US population benchmarks. Our sample was well balanced on most demographic variables but underrepresented Hispanics and overrepresented higher-educated people. Beyond demographics, Republicans were somewhat underrepresented and independents were overrepresented compared to the general population. However, by conducting most of our analyses on the whole sample and on partisan subgroups, we were able to show heterogeneity of effects between these populations.

To determine the required sample size for our study, we conducted two power calculations: one with the R package cjpowR written by Julian Schessler and Markus Freitag and a simulation-based power calculation with the R package DeclareDesign (62). We estimated AMCE effect sizes for two types of analyses: Within each scenario, we postulated an expected effect size at 0.05 and for all scenarios combined (where topic was treated as an additional attribute with four levels) at 0.02. Our power analyses were part of our OSF preregistration at https://osf.io/5g8aq.

### Study Design.

We used a single-profile conjoint survey experiment (41) to explore influences on people’s willingness to remove false and misleading content on social media and penalize the offending accounts. In the main study task, participants saw 16 cases each (Fig. 1). After excluding missing values in responses, this amounted to a total of 40,845 random cases (*SI Appendix*, Table S3; the conjoint design yielded 1,728 possible unique cases).

#### Scenarios.

Each scenario represented a moral dilemma between freedom of expression and potential harm from misinformation. The four scenario types represented four misinformation topics: politics (election denial), health (antivaccination), history (Holocaust denial), and environment (climate change denial), with consequences adjusted for each topic.

#### Attributes in the scenarios.

Each scenario included seven attributes: 1) person (i.e., who shared information; referred to as “account” in the figures); 2) person’s partisanship (“account’s partisanship”); 3) number of followers; 4) action (“misinformation topic”); 5) level of falseness; 6) pattern of behavior; and 7) consequences (“severity of harm”). Each attribute had multiple levels (*SI Appendix*, Table S2; for the distribution of attribute levels, *SI Appendix*, Table S3).

#### Outcome measures.

Respondents were asked to imagine that they had to decide whether to remove the posts mentioned in the scenarios and whether to suspend the account that posted them. These questions represent two dependent variables: choice to remove posts and rating to penalize account. For choice to remove posts, respondents were asked “What would you do with the posts?” and could answer “remove the posts” or “do nothing.” For rating to penalize account, respondents were asked “What would you do with this user’s account?” and could answer “suspend the account indefinitely,” “suspend the account temporarily,” “issue a warning,” or “do nothing” (Fig. 1). Each participant saw 16 cases (four variations of each of the four scenario types) and gave two responses for each (32 responses in total).

#### Attention check.

A simple attention check was presented at the start of the study: Participants were asked, “How many scenarios are you expected to see?” The question was displayed on the same page as the description of the main task, which included the correct answer (16) in bold characters. Participants who did not pass the attention check were redirected to the study termination page. This information was included in the consent form.

#### Demographics and political attitudes.

After consent but before the main study task, respondents filled out demographic information and information on their political attitudes (*SI Appendix*, Table S1).

#### Perceived accuracy, harm, and severity of outcomes.

After the conjoint task, respondents rated the accuracy of a statement related to each topic of misinformation (“The 2020 U.S. Presidential election was stolen from Donald Trump,” “The FDA-approved COVID-19 vaccines can cause infertility,” “The death of 6 million Jews in the Holocaust is a well established historical fact,” and “There is an overwhelming scientific consensus that human activity, e.g., burning fossil fuels is the leading cause of climate change”) on a 5-point Likert scale (definitely false, probably false, don’t know, probably true, and definitely true; *SI Appendix*, Fig. S13). Respondents also rated the perceived harm of the content featured in each scenario on a 5-point Likert scale (not at all harmful, a little harmful, somewhat harmful, very harmful, and extremely harmful; *SI Appendix*, Fig. S14) and the perceived severity of the outcomes featured in each scenario on a 5-point Likert scale (not severe at all, slightly severe, somewhat severe, very severe, and extremely severe; *SI Appendix*, Fig. S15).

#### Attitudes toward freedom of expression.

We included measures of people’s attitudes toward freedom of expression and its limitations. Four questions addressed participants’ general attitudes toward freedom of expression and its limits in cases of prejudice, falsehoods, and potential for harm (four items adapted from ref. 63; for items and distribution of responses, *SI Appendix*, Fig. S12). Two further questions addressed people’s preferences in the dilemma between freedom of expression and preventing harmful misinformation: One asked participants to choose between freedom of expression and preventing disinformation from spreading and another asked them to choose between a hypothetical social media platform that always prioritizes free speech and another that moderates content strictly. Participants answered these two questions both before and after the main study task so that we could compare proportions of respondents who were willing to impose limits on free expression to mitigate harmful misinformation before and after they faced the moral dilemmas in our scenarios (for items and distribution of responses, *SI Appendix*, Figs. S10 and S11).

#### Estimates of N of misinformation accounts.

We administered one item after the second set of questions on attitudes toward freedom of expression. This item asked participants to estimate how many accounts produce the majority of disinformation on social media (“To the best of your knowledge, how many individuals are responsible for 65% of the antivaccination disinformation on Facebook and Twitter? Please indicate or estimate a number.”). We based the correct answer on the Center for Countering Digital Hate’s recent estimate that 12 accounts are responsible for 65% of the antivaccination misinformation on Facebook and Twitter (64). For results, *SI Appendix*, Fig. S16.

The full instrument is available on OSF at https://osf.io/2s4vn/.

### Data Analysis.

We report both descriptive and inferential statistics. For descriptive analyses, we reported the demographic distribution of our sample, frequencies of conjoint features, and proportions of choices for several measures in the study.

The main analysis was the conjoint analysis used to estimate causal effects of multiple factors (attributes) on the binary decision to remove posts in all four scenarios and the rating measure on whether to suspend an account (permanently or temporarily), issue a warning, or do nothing; we report both results for a dichotomized version of this rating (do nothing/issue a warning vs. temporarily/permanently suspend the account).

We conducted the main analysis using cregg (65), an R package for analyzing and visualizing the results of conjoint experiments. Although our preregistration stated that we would use cjoint (66), cregg’s superior functionality for our purposes justified this choice. We reported on estimates for two estimands (41, 42, 67): marginal means and AMCEs. Marginal means facilitate interpretations of conjoint attributes’ impact on respondents’ decisions not predicated on a specific reference category, whereas AMCEs show effect sizes relative to the chosen reference levels (67).

### Preregistration.

The study was preregistered at OSF (https://osf.io/5g8aq). The preregistration also includes the full study instrument and the power analysis. Analyses of all measures included in the study and of some preregistered research questions that did not appear in the main text are provided in *SI Appendix*, *Appendix B*. These additional results do not alter any of the results or conclusions presented in the main text.

### Ethics.

Informed consent was obtained from all participants, and the study was conducted in accordance with relevant guidelines and regulations. The Institutional Review Board of the Max Planck Institute for Human Development approved the study (approval C2021-16).

## Supplementary Material

Appendix 01 (PDF)

## Data Availability

Anonymized data and code are available at OSF (https://osf.io/2s4vn/).
