# Supplementary material for: Resolving content moderation dilemmas between free speech and harmful misinformation
Source: Proc Natl Acad Sci U S A. 2023 Feb 7;120(7):e2210666120. doi: 10.1073/pnas.2210666120 (PMC9963596; doi:10.1073/pnas.2210666120)
Supplement: Supplementary file 1 — Appendix 01 (PDF) [file pnas.2210666120.sapp.pdf]

Supporting Information for

Resolving content moderation dilemmas between free speech and harmful misinformation

Anastasia Kozyreva, Stefan M. Herzog, Stephan Lewandowsky, Ralph Hertwig, Philipp Lorenz-Spreen, Mark Leiser, and Jason Reifler

Jason Reifler.
Email: j.reifler@exeter.ac.uk

This PDF file includes:

Figs. S1 to S19
Tables S1 to S10
SI References

Appendix A: Methods and Conjoint Analyses

Table S1. Sample Information

	study sample	benchmark
**Sample size**		
N	2564.0	NA
**Duration (minutes)**		
Duration (mean)	21.0	NA
Duration (median)	13.0	NA
**Gender (%)**		
Male	48.5	48.3
Female	51.2	51.7
Other	0.3	NA
**Age group (%)**		
Age (18-24)	9.4	10.9
Age (25-34)	16.9	18.0
Age (35-44)	17.1	16.7
Age (45-54)	16.7	16.2
Age (55-64)	17.6	16.9
Age (65+)	22.4	21.2
**Education (%)**		
Less than high school	2.7	11.2
High school	27.0	27.3
Some college	19.9	21.5
Associate or BA degree	34.4	28.2
Master's degree	13.1	8.3
Doctoral degree	3.0	3.3
**Region of residency (%)**		
South	36.6	38.0
West	23.2	23.8
Northeast	19.7	17.3
Midwest	20.4	20.7
**Ethnicity (%)**		
Other	2.8	2.4
White	70.8	63.2
Black or African-American	12.8	11.8
Hispanic or Latino	6.4	16.4
Asian or Asian-American	7.2	5.9
**Political party, 7-point scale (%)**		
Strong Democrat	24.9	23.0
Moderate Democrat	13.7	12.0
Lean Democrat	8.7	11.0
Independent	14.4	12.0
Lean Republican	6.3	10.0
Moderate Republican	10.5	11.0
Strong Republican	15.8	21.0
Not sure	5.7	NA
**Political party, 3-point scale (%)**		
Democrats and democratic leaners	47.3	46.0
Independent or not sure	20.1	12.0
Republicans or republican leaners	32.5	42.0
**Political ideology(%)**		
Liberal	29.6	27.0
Moderate	35.7	22.0
Conservative	27.8	33.0
Not sure	6.9	17.0

Benchmarks for demographic variables: American Community Survey 2019.
Benchmarks for political party identification and ideology: The ANES Guide to Public Opinion and Electoral Behavior (2020).

Table S2. Conjoint Table

Attribute	Levels	N levels
Person (Account)	"an elected politician", "a political activist", "a celebrity", "a private citizen"	4
Person (Account) for the "Election denial" scenario	"a presidential candidate", "a political activist", "a celebrity", "a private citizen"	4
Person's partisanship (Account's partisanship)	"who is a Democrat", "who is a Republican", "who is an independent"	3
N of followers	"with less than 100,000 followers on a popular social media platform," "with about 500,000 followers on a popular social media platform," "with more than 1 million followers on a popular social media platform,"	3
Action (Misinformation topic) 1 ("Election denial" scenario)	"published a series of posts denying the outcome of the presidential election, encouraging people to join a protest rally and praising violent supporters."	1
Action (Misinformation topic) 2 ("Anti-vaccination" scenario)	"published a series of posts about serious side effects of the approved COVID-19 vaccines (e.g., that vaccines cause infertility)."	1
Action (Misinformation topic) 3 ("Holocaust denial" scenario)	"published a series of posts questioning the scale of the Holocaust (e.g., that significantly fewer than 6 million Jews were killed)."	1
Action (Misinformation topic) 4 ("Climate change denial" scenario)	"published a series of posts denying scientific consensus that human activity (e.g., burning fossil fuels) is the leading cause of climate change."	1
Level of falseness	"The specific information they shared is completely false and negates the established facts."; "The specific information they shared is misleading and distorts the established facts."	2
Pattern of behavior	"This was the first time they shared false or misleading information.", "This was not the first time they shared false or misleading information."	2
Consequences (Severity of harms) 1 ("Election denial" scenario)	No consequences: "Suppose you know that these messages caused no consequences."; Medium: "Suppose you know that, due to this, a nonviolent demonstration occurred."; Severe: "Suppose you know that, due to this, a violent demonstration occurred, 5 people died, and 150 protesters were detained."	3
Consequences (Severity of harms) 2 ("Anti-vaccination" scenario)	"Suppose you know that these messages caused no consequences.", "Suppose you know that, due to this, 10,000 citizens who were planning to get a vaccine refused to vaccinate.", "Suppose you know that, due to this, 1 million people who were planning to get a vaccine refused to vaccinate, resulting in approximately 10,000 additional deaths."	3
Consequences (Severity of harms) 3 ("Holocaust denial" scenario)	"Suppose you know that these messages caused no consequences.", "Suppose you know that, due to this, several antisemitic attacks occurred, with no severe injuries.", "Suppose you know that, due to this, several antisemitic attacks occurred, injuring 2 people and killing 1 person."	3
Consequences (Severity of harms) 4 ("Climate change denial" scenario)	"Suppose you know that these messages caused no consequences.", "Suppose you know that these posts convinced 1,000 people that climate change is a hoax.", "Suppose you know that these posts convinced 100,000 voters that climate change is a hoax, thereby swinging the outcome of the next election and preventing the passage of a bill that would have cut carbon emissions by 20%."	3

Table S3. Frequency of Conjoint Features

**Attribute** and Levels	N	%
**Account**		
Private citizen	9,558	23.4
Celebrity	10,789	26.4
Political activist	10,804	26.5
Politician	9,694	23.7
**Account's partisanship**		
Independent	14,453	35.4
Democrat	12,618	30.9
Republican	13,774	33.7
**N of followers**		
< 100,000	12,820	31.4
~ 500,000	12,835	31.4
> 1,000,000	15,190	37.2
**Action/Misinformation topic**		
Climate change denial	10,256	25.1
Holocaust denial	10,077	24.7
Anti-vaccination	10,256	25.1
Election denial	10,256	25.1
**Level of falseness**		
Misleading	20,957	51.3
Completely false	19,888	48.7
**Pattern of behavior**		
First time	20,249	49.6
Repeated	20,596	50.4
**Consequences/Severity of harms**		
None	13,685	33.5
Medium	13,380	32.8
Severe	13,780	33.7
**Total N per attribute**		
	40,845	

Table S4. AMCEs for choice to remove post

	Attribute	Level	Estimate	SE	Lower CI	Upper CI
1	Misinformation topic	Climate change denial	0.00			
2	Misinformation topic	Holocaust denial	0.13	0.01	0.11	0.14
3	Misinformation topic	Anti-vaccination	0.08	0.01	0.07	0.09
4	Misinformation topic	Election denial	0.11	0.01	0.10	0.12
5	Severity of harms	None	0.00			
6	Severity of harms	Medium	0.04	0.01	0.02	0.06
7	Severity of harms	Severe	0.09	0.01	0.07	0.11
8	Pattern of behavior	First time	0.00			
9	Pattern of behavior	Repeated	0.04	0.01	0.02	0.06
10	Information’s falseness	Misleading	0.00			
11	Information’s falseness	Completely false	0.02	0.01	0.00	0.03
12	Account	Private citizen	0.00			
13	Account	Celebrity	0.01	0.01	-0.02	0.03
14	Account	Political activist	0.02	0.01	-0.01	0.04
15	Account	Politician	0.02	0.01	-0.01	0.04
16	Account’s partisanship	Independent	0.00			
17	Account’s partisanship	Democrat	0.01	0.01	-0.01	0.03
18	Account’s partisanship	Republican	-0.00	0.01	-0.02	0.02
19	Number of followers	$<$ 100,000	0.00			
20	Number of followers	~ 500,000	-0.01	0.01	-0.03	0.01
21	Number of followers	$>$ 1,000,000	-0.00	0.01	-0.03	0.02

SE = standard error; CI = confidence interval.

Table S5. AMCEs for Rating to Penalize Account

	Attribute	Level	Estimate	SE	Lower CI	Upper CI
1	Misinformation topic	Climate change denial	0.00			
2	Misinformation topic	Holocaust denial	0.36	0.01	0.34	0.39
3	Misinformation topic	Anti-vaccination	0.21	0.01	0.19	0.23
4	Misinformation topic	Election denial	0.36	0.01	0.33	0.38
5	Severity of harms	None	0.00			
6	Severity of harms	Medium	0.15	0.02	0.11	0.19
7	Severity of harms	Severe	0.41	0.02	0.36	0.45
8	Pattern of behavior	First time	0.00			
9	Pattern of behavior	Repeated	0.30	0.02	0.26	0.34
10	Information's falseness	Misleading	0.00			
11	Information's falseness	Completely false	0.04	0.02	0.01	0.08
12	Account	Private citizen	0.00			
13	Account	Celebrity	0.02	0.02	-0.03	0.07
14	Account	Political activist	0.05	0.02	0.01	0.10
15	Account	Politician	0.05	0.03	0.00	0.10
16	Account's partisanship	Independent	0.00			
17	Account's partisanship	Democrat	0.03	0.02	-0.01	0.07
18	Account's partisanship	Republican	0.02	0.02	-0.02	0.06
19	Number of followers	$<$ 100,000	0.00			
20	Number of followers	~ 500,000	0.03	0.02	-0.01	0.07
21	Number of followers	$>$ 1,000,000	0.03	0.02	-0.02	0.07

SE = standard error; CI = confidence interval.

Table S6. AMCEs for Binarized Rating to Penalize Account

	Attribute	Level	Estimate	SE	Lower CI	Upper CI
1	Misinformation topic	Climate change denial	0.00			
2	Misinformation topic	Holocaust denial	0.16	0.01	0.14	0.17
3	Misinformation topic	Anti-vaccination	0.09	0.01	0.08	0.10
4	Misinformation topic	Election denial	0.15	0.01	0.14	0.16
5	Severity of harms	None	0.00			
6	Severity of harms	Medium	0.08	0.01	0.06	0.10
7	Severity of harms	Severe	0.19	0.01	0.17	0.21
8	Pattern of behavior	First time	0.00			
9	Pattern of behavior	Repeated	0.17	0.01	0.16	0.19
10	Information's falseness	Misleading	0.00			
11	Information's falseness	Completely false	0.02	0.01	0.01	0.04
12	Account	Private citizen	0.00			
13	Account	Celebrity	0.01	0.01	-0.01	0.03
14	Account	Political activist	0.02	0.01	-0.00	0.04
15	Account	Politician	0.02	0.01	-0.00	0.04
16	Account's partisanship	Independent	0.00			
17	Account's partisanship	Democrat	0.02	0.01	-0.00	0.04
18	Account's partisanship	Republican	0.01	0.01	-0.01	0.02
19	Number of followers	$<$ 100,000	0.00			
20	Number of followers	˜ 500,000	0.02	0.01	-0.00	0.04
21	Number of followers	$>$ 1,000,000	0.02	0.01	0.00	0.04

SE = standard error; CI = confidence interval.

Table S7. Marginal Means for Choice to Remove Post

	Attribute	Level	Estimate	SE	Lower CI	Upper CI
1	Misinformation topic	Climate change denial	0.58	0.01	0.56	0.60
2	Misinformation topic	Holocaust denial	0.71	0.01	0.69	0.72
3	Misinformation topic	Anti-vaccination	0.66	0.01	0.64	0.68
4	Misinformation topic	Election denial	0.69	0.01	0.67	0.70
5	Severity of harms	None	0.61	0.01	0.60	0.63
6	Severity of harms	Medium	0.66	0.01	0.64	0.67
7	Severity of harms	Severe	0.70	0.01	0.68	0.72
8	Pattern of behavior	First time	0.64	0.01	0.62	0.66
9	Pattern of behavior	Repeated	0.67	0.01	0.66	0.69
10	Information's falseness	Misleading	0.65	0.01	0.63	0.67
11	Information's falseness	Completely false	0.67	0.01	0.65	0.68
12	Account	Private citizen	0.65	0.01	0.63	0.67
13	Account	Celebrity	0.65	0.01	0.63	0.67
14	Account	Political activist	0.67	0.01	0.65	0.69
15	Account	Politician	0.67	0.01	0.65	0.69
16	Account's partisanship	Independent	0.65	0.01	0.63	0.67
17	Account's partisanship	Democrat	0.67	0.01	0.65	0.69
18	Account's partisanship	Republican	0.65	0.01	0.64	0.67
19	Number of followers	$<$ 100,000	0.66	0.01	0.64	0.68
20	Number of followers	~ 500,000	0.65	0.01	0.64	0.67
21	Number of followers	$>$ 1,000,000	0.66	0.01	0.64	0.68

SE = standard error; CI = confidence interval.

Table S8. Marginal Means for Rating to Penalize Account

	Attribute	Level	Estimate	SE	Lower CI	Upper CI
1	Misinformation topic	Climate change denial	2.18	0.02	2.15	2.22
2	Misinformation topic	Holocaust denial	2.55	0.02	2.51	2.58
3	Misinformation topic	Anti-vaccination	2.39	0.02	2.36	2.43
4	Misinformation topic	Election denial	2.54	0.02	2.51	2.58
5	Severity of harms	None	2.24	0.02	2.20	2.28
6	Severity of harms	Medium	2.38	0.02	2.34	2.42
7	Severity of harms	Severe	2.62	0.02	2.58	2.67
8	Pattern of behavior	First time	2.28	0.02	2.24	2.31
9	Pattern of behavior	Repeated	2.55	0.02	2.51	2.59
10	Information's falseness	Misleading	2.41	0.02	2.38	2.45
11	Information's falseness	Completely false	2.42	0.02	2.38	2.45
12	Account	Private citizen	2.36	0.02	2.32	2.41
13	Account	Celebrity	2.36	0.02	2.32	2.40
14	Account	Political activist	2.48	0.02	2.44	2.53
15	Account	Politician	2.45	0.02	2.41	2.49
16	Account's partisanship	Independent	2.38	0.02	2.34	2.42
17	Account's partisanship	Democrat	2.44	0.02	2.40	2.48
18	Account's partisanship	Republican	2.44	0.02	2.39	2.48
19	Number of followers	$<$ 100,000	2.37	0.02	2.33	2.41
20	Number of followers	~ 500,000	2.42	0.02	2.38	2.46
21	Number of followers	$>$ 1,000,000	2.45	0.02	2.41	2.49

SE = standard error; CI = confidence interval.

Table S9. Marginal Means for Binarized Rating to Penalize Account

	Attribute	Level	Estimate	SE	Lower CI	Upper CI
1	Misinformation topic	Climate change denial	0.35	0.01	0.33	0.36
2	Misinformation topic	Holocaust denial	0.50	0.01	0.49	0.52
3	Misinformation topic	Anti-vaccination	0.44	0.01	0.43	0.46
4	Misinformation topic	Election denial	0.50	0.01	0.48	0.51
5	Severity of harms	None	0.36	0.01	0.35	0.38
6	Severity of harms	Medium	0.44	0.01	0.42	0.46
7	Severity of harms	Severe	0.54	0.01	0.52	0.56
8	Pattern of behavior	First time	0.37	0.01	0.35	0.38
9	Pattern of behavior	Repeated	0.53	0.01	0.51	0.54
10	Information's falseness	Misleading	0.45	0.01	0.43	0.46
11	Information's falseness	Completely false	0.45	0.01	0.43	0.46
12	Account	Private citizen	0.42	0.01	0.40	0.44
13	Account	Celebrity	0.42	0.01	0.41	0.44
14	Account	Political activist	0.48	0.01	0.46	0.49
15	Account	Politician	0.47	0.01	0.45	0.49
16	Account's partisanship	Independent	0.43	0.01	0.41	0.44
17	Account's partisanship	Democrat	0.46	0.01	0.44	0.48
18	Account's partisanship	Republican	0.46	0.01	0.44	0.48
19	Number of followers	$<$ 100,000	0.42	0.01	0.41	0.44
20	Number of followers	˜ 500,000	0.45	0.01	0.43	0.46
21	Number of followers	$>$ 1,000,000	0.47	0.01	0.45	0.49

SE = standard error; CI = confidence interval.

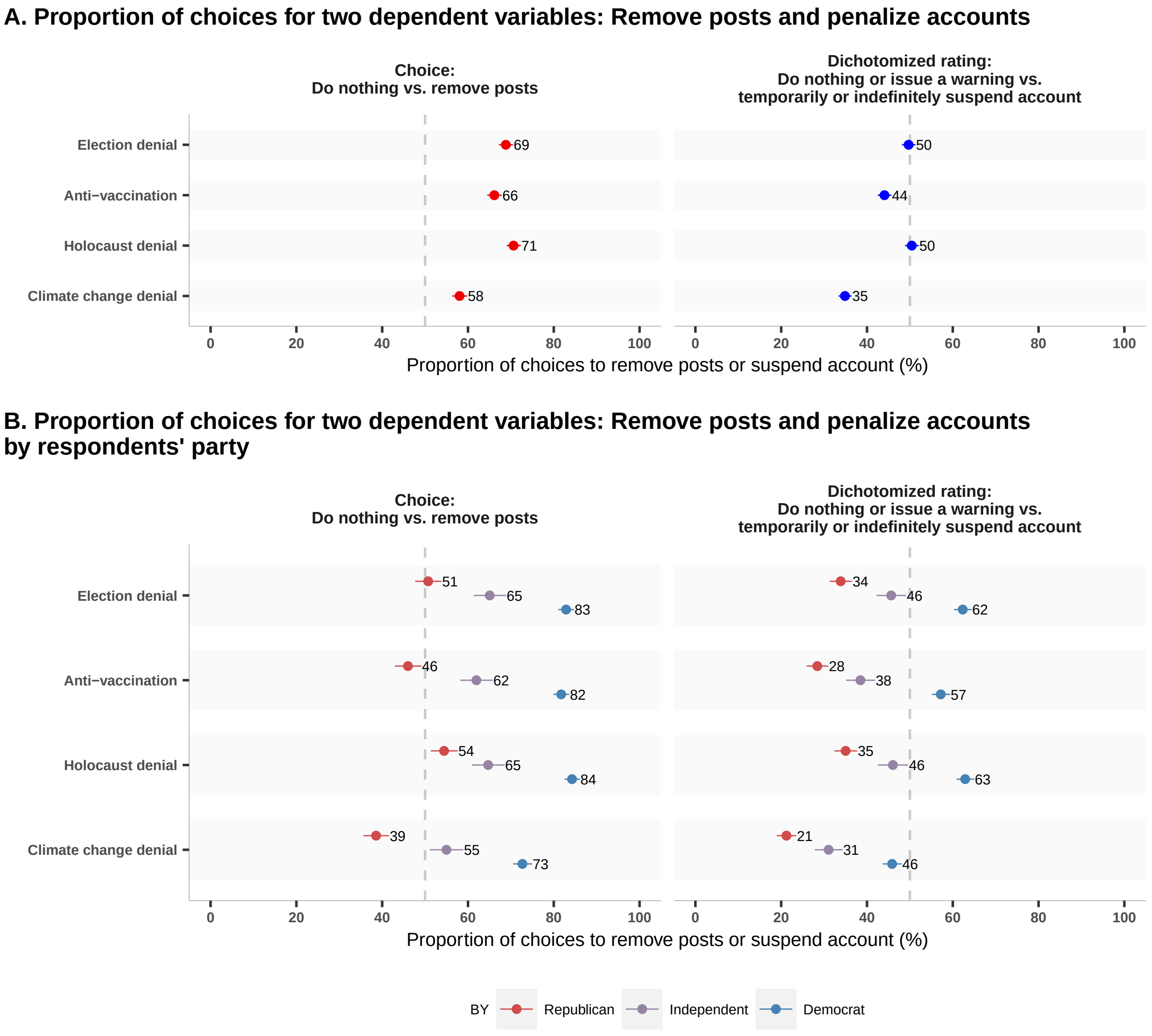

Fig. S1. *Proportion of choices to remove posts and to suspend accounts.* All numeric values represent percentages and are plotted with 95% confidence intervals. Panel A: Choices to remove posts or do nothing by misinformation topic (all cases) for two dependent variables: binary choice to remove the posts vs. do nothing and dichotomized rating to do nothing/issue a warning vs. temporarily/indefinitely suspend. Panel B: Choices to penalize account by topic and respondents' party affiliation. $N = 40,845$ evaluated cases in total. (Cases evaluated by Democrats $n = 19,338$; by independents $n = 8,229$; by Republicans $n = 13,278$.) This figure complements Figure 2 in the main text.

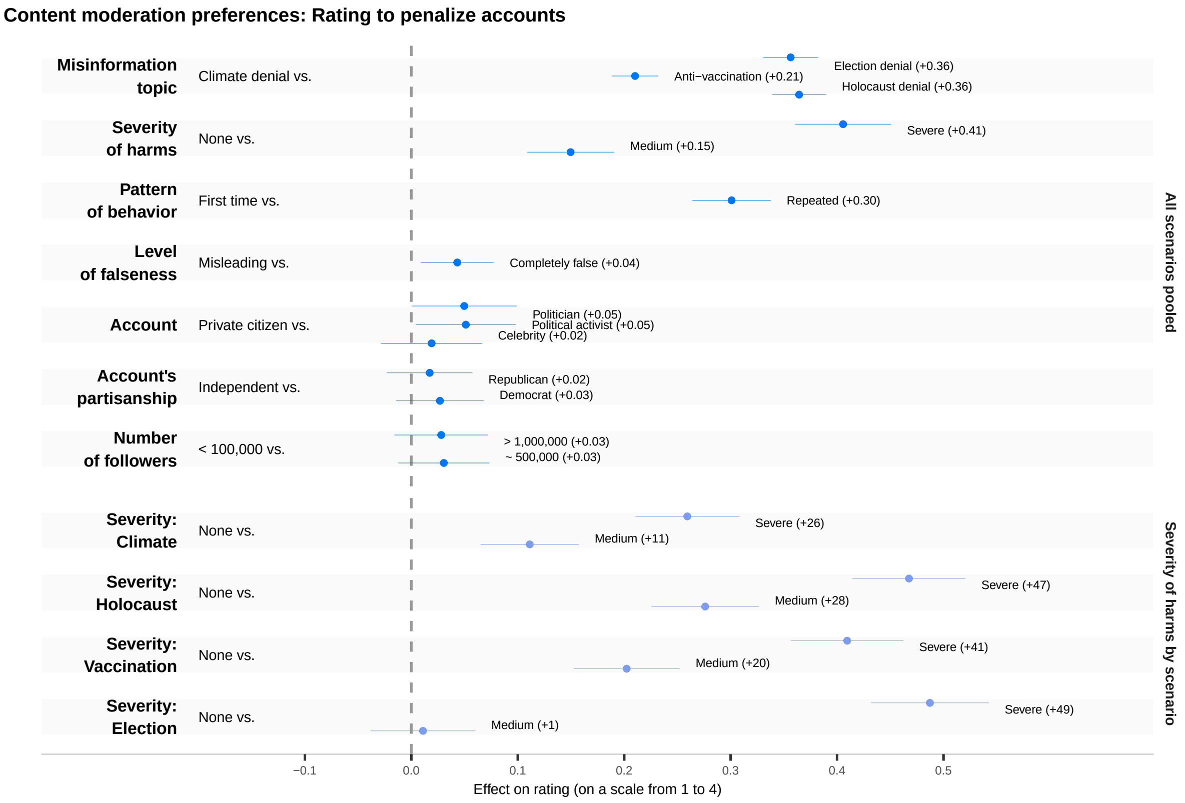

Fig. S2. *Preferences for content moderation: Rating.* The figure reports average marginal component effects (AMCEs) plotted with 95% confidence intervals. In each row, effect sizes show an impact of each attribute level (on the right) relative to the reference attribute level (on the left), aggregated over all other attributes. In both panels, “all scenarios pooled” displays all attributes, including severity of harms, in a pooled manner. As, in each scenario, the consequences were matched to the respective misinformation topic (and thus, unlike all other attributes, were not common across topics), “severity of harms by scenario” shows scenario-specific effects for this attribute. This figure complements Figure 3 in the main text.

A. Marginal means by scenario: Choice to remove posts

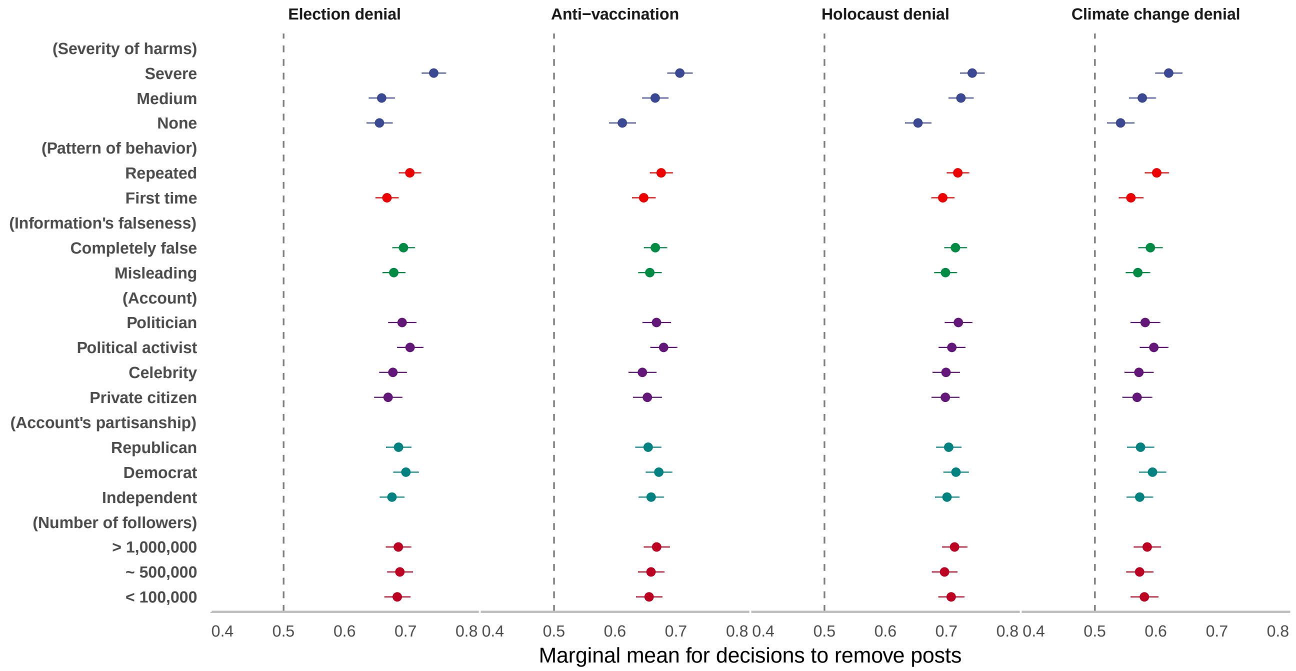

B. AMCEs by scenario: Choice to remove posts

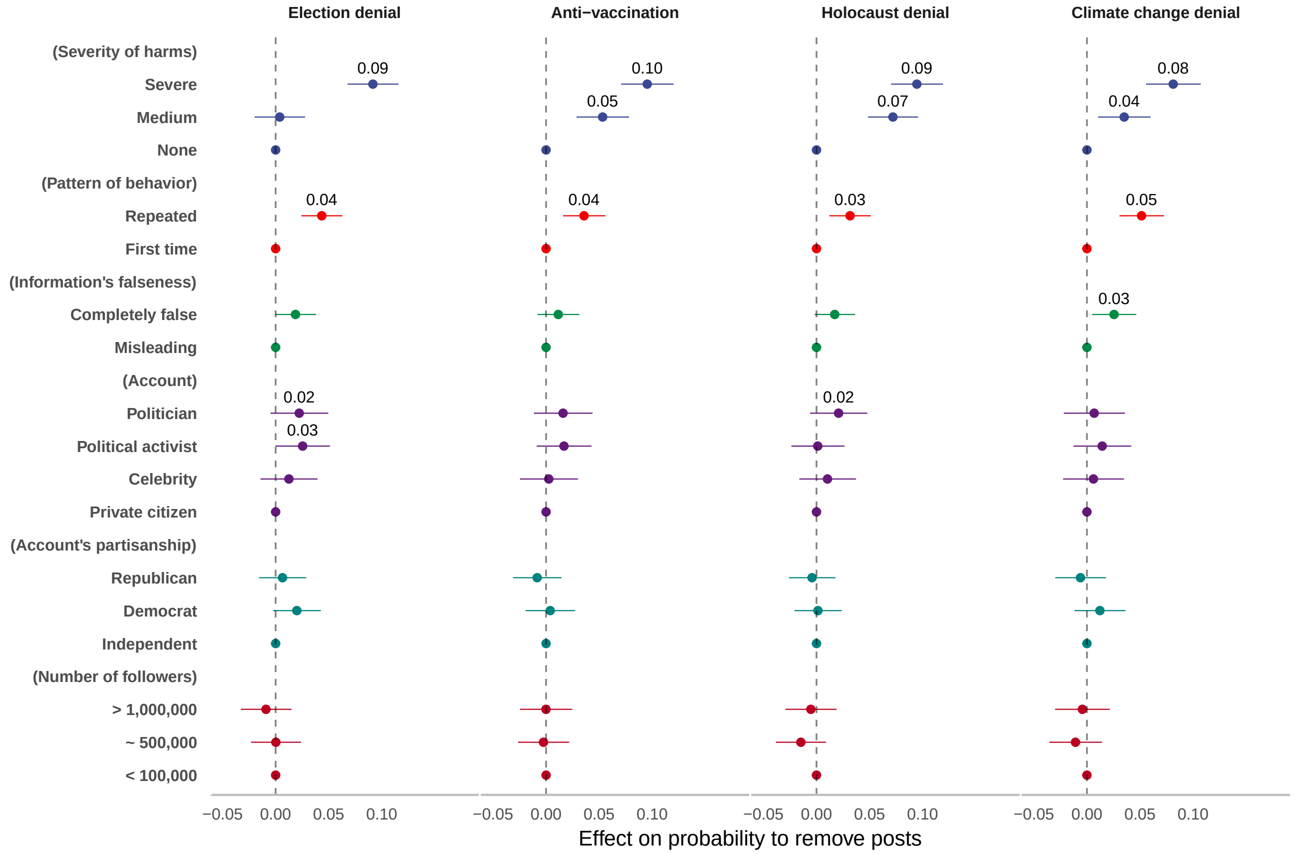

Fig. S3. *Marginal means and average marginal component effects (AMCEs) for choices to remove the post for each scenario type.* Marginal means point estimates and AMCEs plotted with 95% confidence intervals. Panel A: Marginal means represent the average likelihood of decisions to remove the posts for each attribute level faceted by four scenario types. Dashed lines represent the mean value for a binary decision (0.5). Panel B: AMCEs represent effects on probability to remove the posts for each attribute level faceted by four scenario types. Dashed lines represent the null effect.

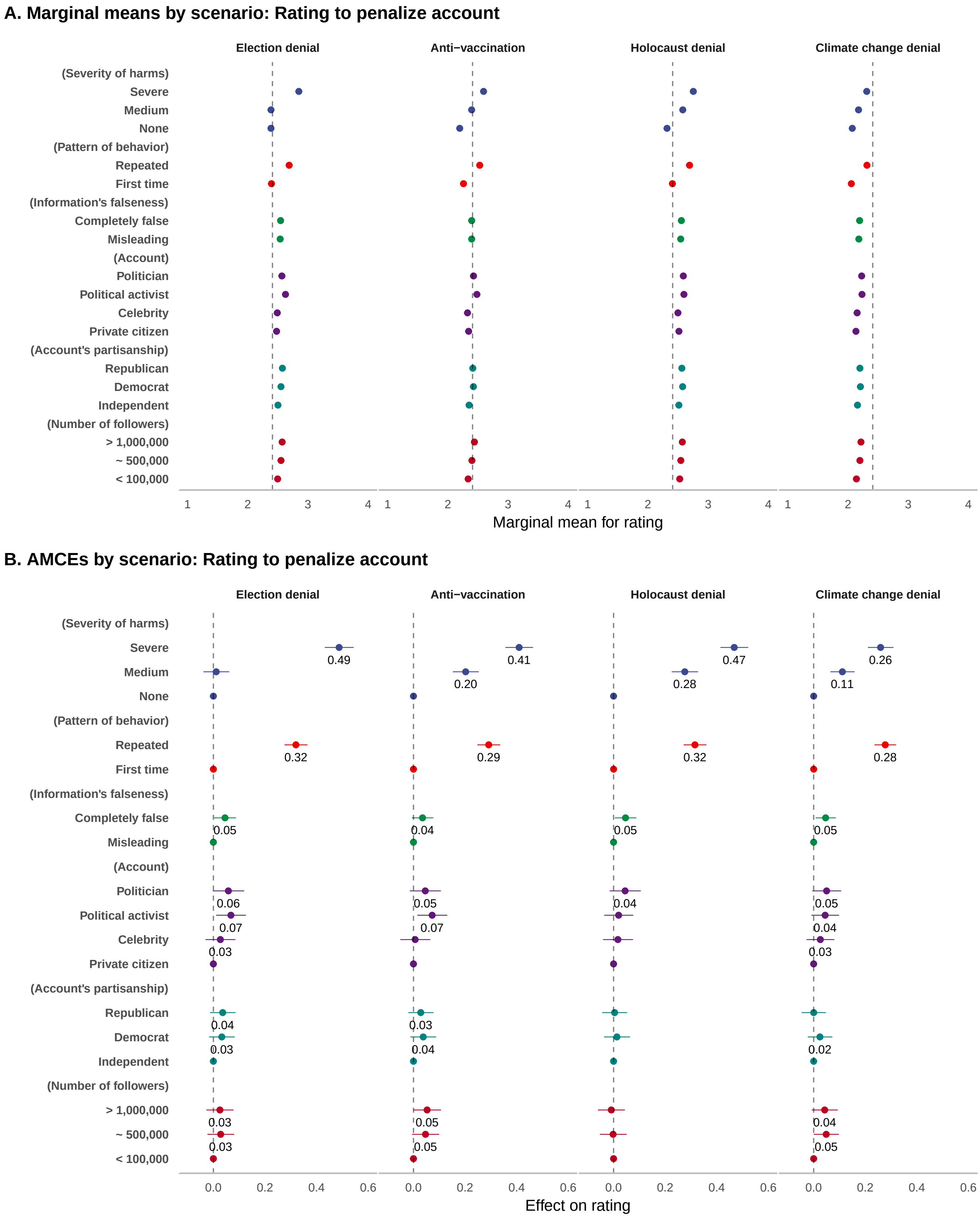

Fig. S4. *Marginal means and average marginal component effects (AMCEs) for ratings to penalize accounts for each scenario type.* Marginal means point estimates and AMCEs plotted with 95% confidence intervals. Panel A: Marginal means represent the average rating for decisions to penalize the account for each attribute level, faceted by four scenario types. Dashed lines represent grand mean for rating (2.41). Panel B: AMCEs represent effects on rating to penalize the account for each attribute level, faceted by four scenario types. Dashed lines represent the null effect.

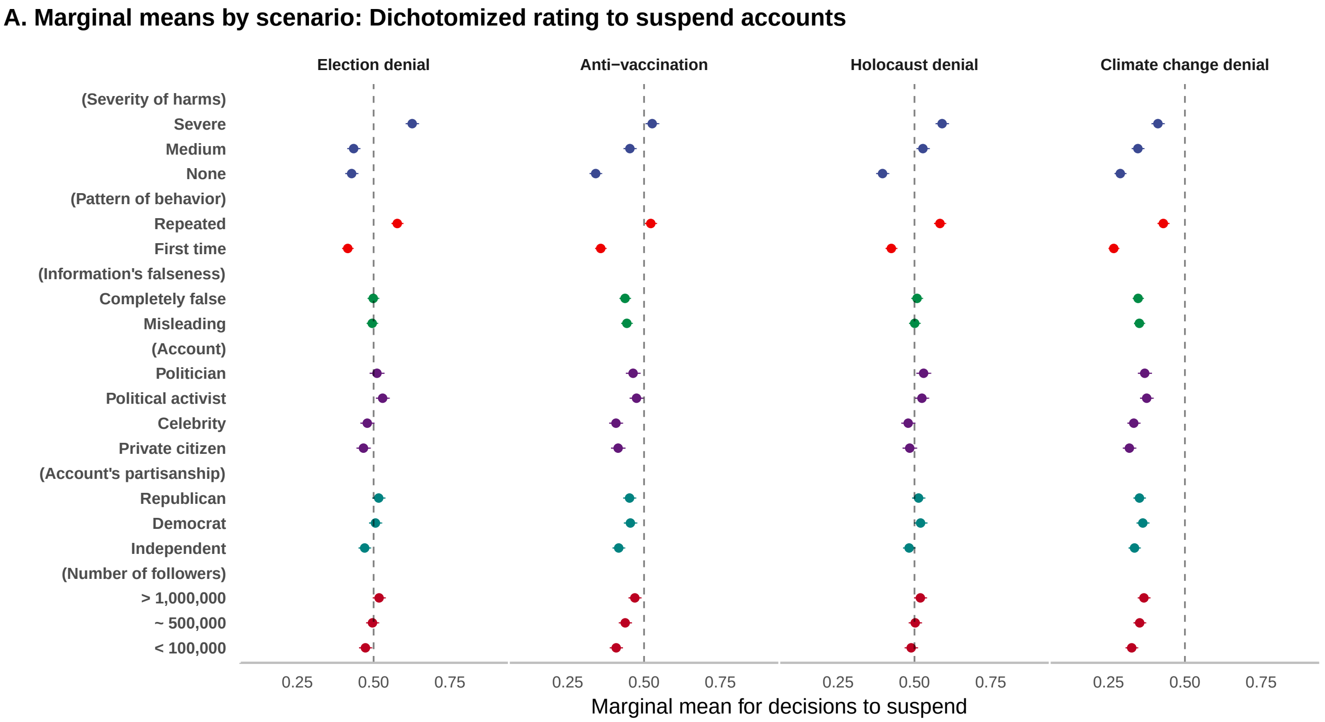

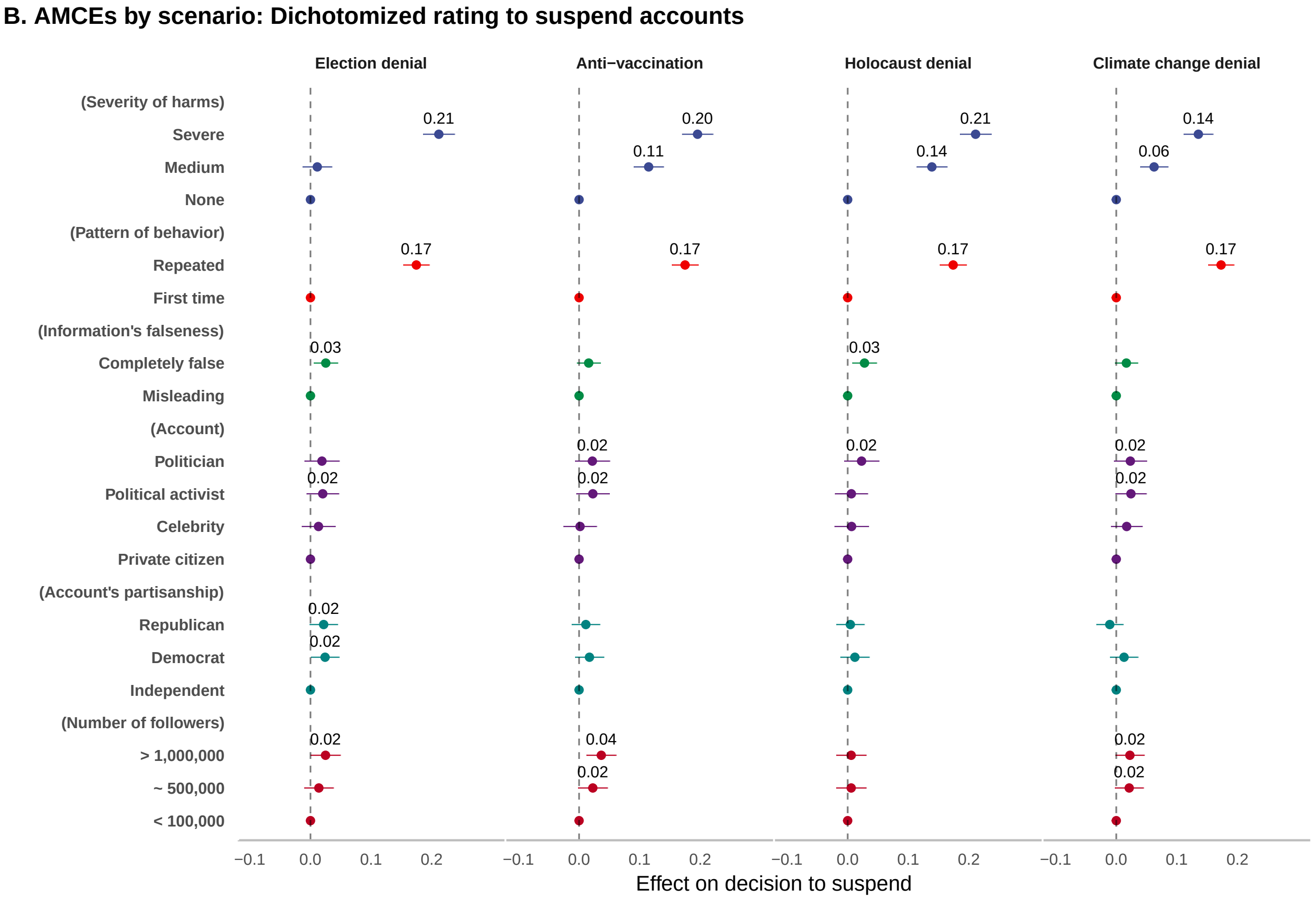

Fig. S5. *Marginal means and average marginal component effects (AMCEs) for dichotomized rating to suspend accounts for each scenario type.* Marginal means point estimates and AMCEs plotted with 95% confidence intervals. Panel A: Marginal means represent the average rating for decisions to penalize the account for each attribute level, faceted by four scenario types. Dashed lines represent the mean value for a binary decision (0.5). Panel B: AMCEs represent effects on rating to penalize the account for each attribute level, faceted by four scenario types. Dashed lines represent the null effect.

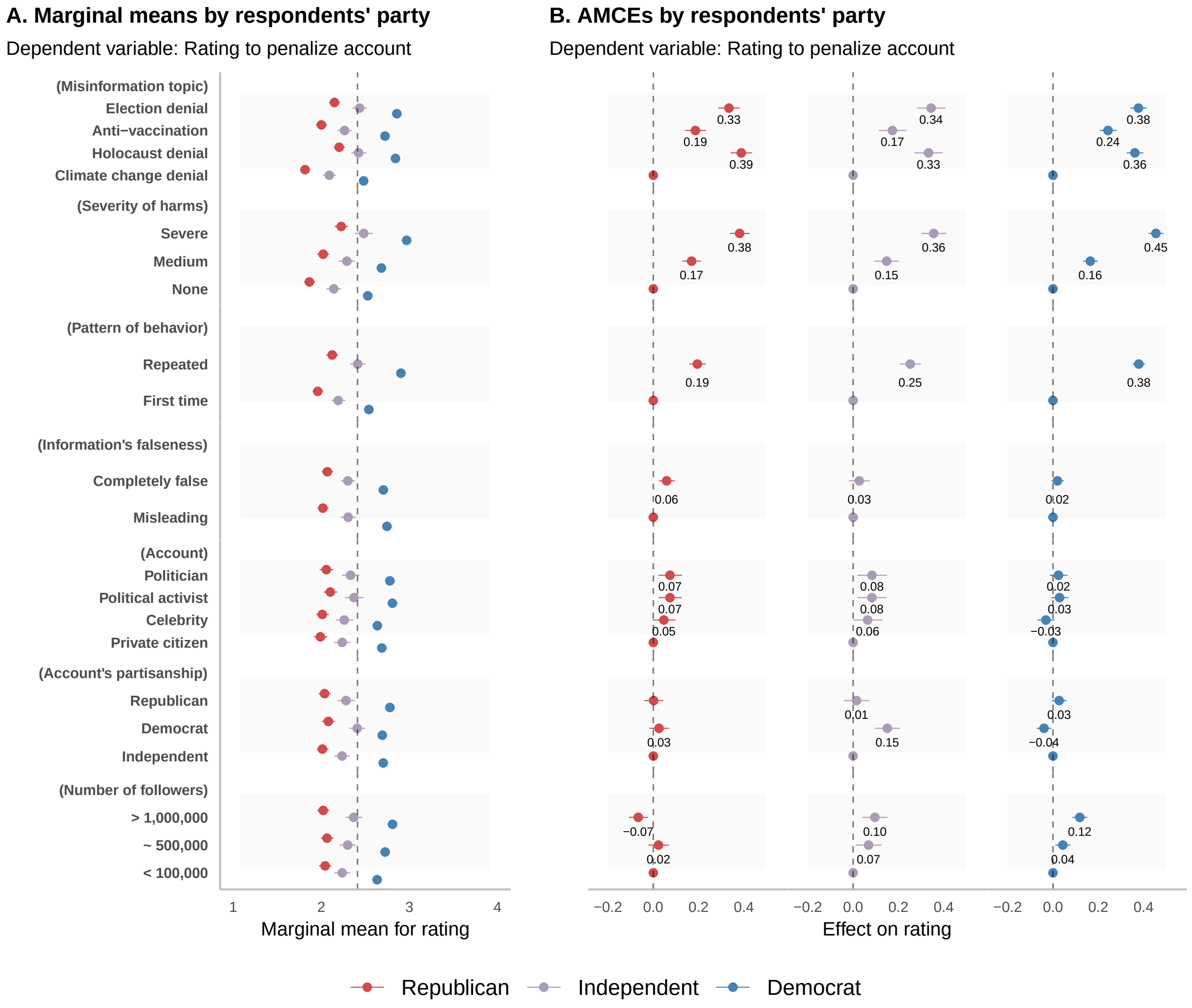

Fig. S6. *Respondent subgroup analyses: Rating by respondents' party affiliation.* Marginal means point estimates and average marginal component effects (AMCEs) plotted with 95% confidence intervals. Panel A: Marginal means represent the average rating for decisions to penalize the account for each attribute level for three respondent subgroups: Republicans, independents, and Democrats. Dashed line represents the grand mean for rating (2.41). Panel B: AMCEs represent effects on rating to penalize the account for each attribute level, faceted by three subgroups: Republicans, independents, and Democrats. Dashed lines represent the null effect.

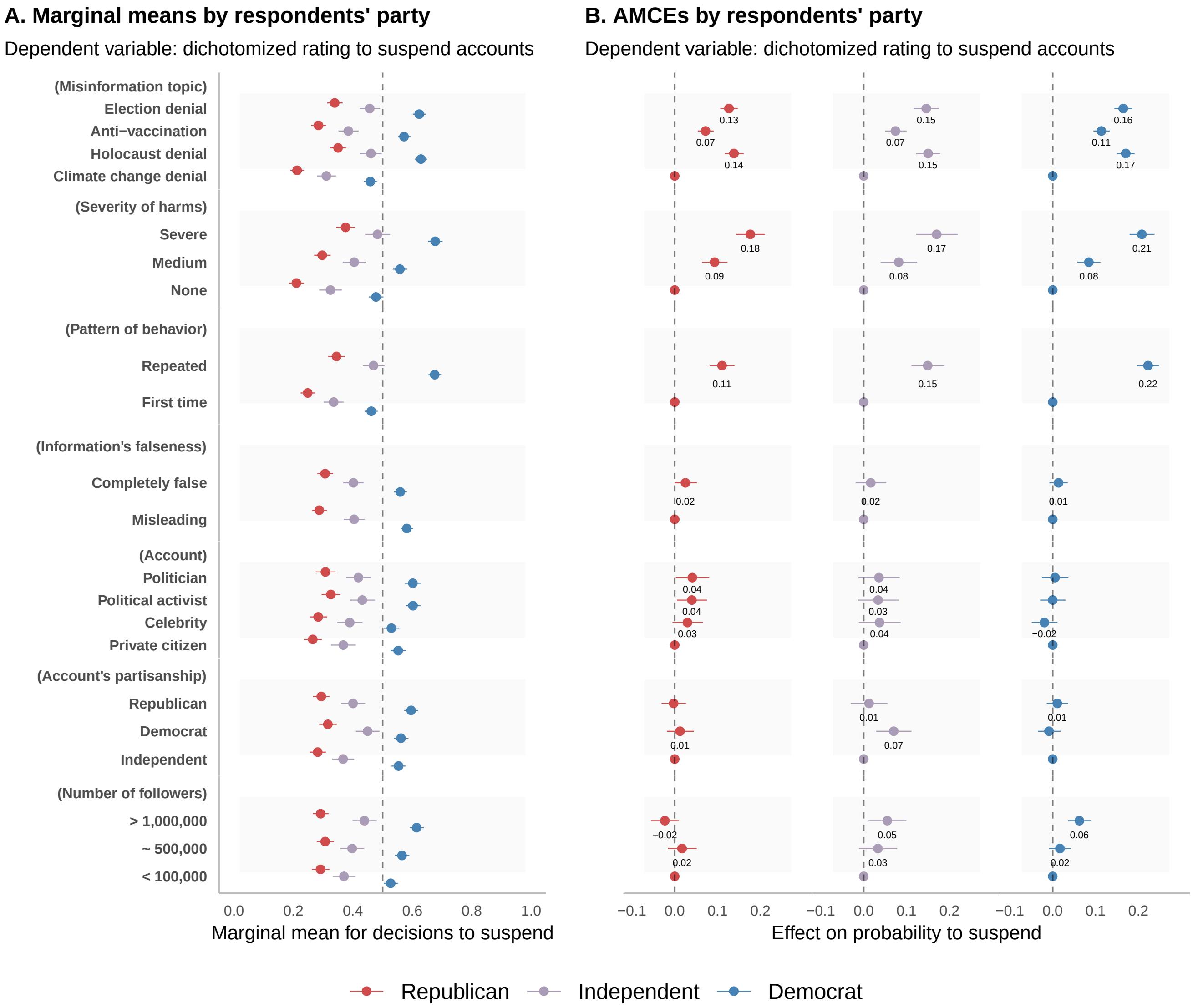

Fig. S7. *Respondent subgroup analyses: Dichotomized rating by respondents' party affiliation.* Marginal means point estimates and average marginal component effects (AMCEs) plotted with 95% confidence intervals. Panel A: Marginal means represent the average rating for decisions to penalize the account for each attribute level for three respondent subgroups: Republicans, independents, and Democrats. Dashed line represents the mean value for a binary decision (0.5). Panel B: AMCEs represent effects on rating to penalize the account for each attribute level, faceted by three subgroups: Republicans, independents, and Democrats. Dashed lines represent the null effect.

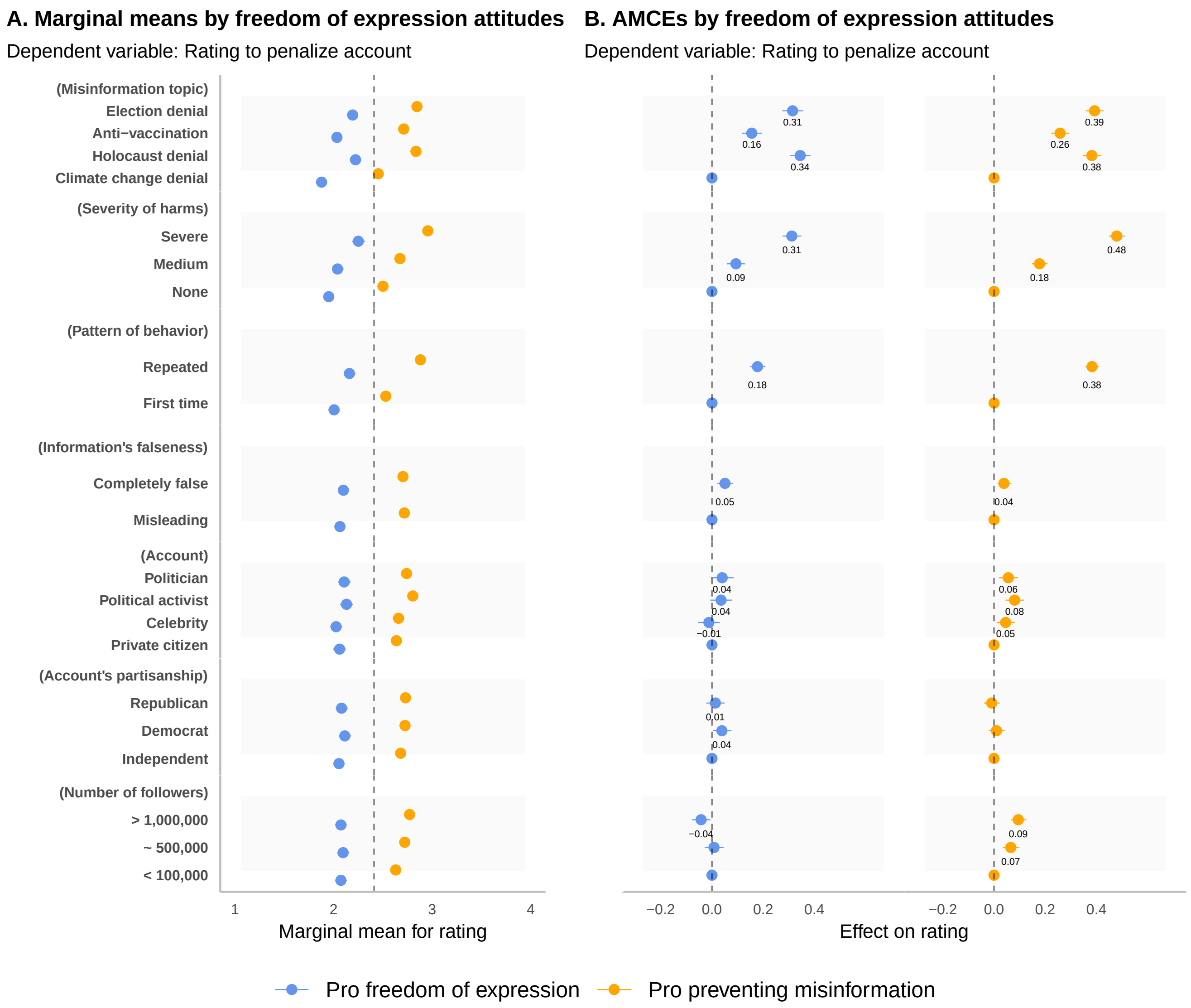

Fig. S8. *Respondent subgroup analyses: Rating by respondents' attitudes toward free speech.* Marginal means point estimates and average marginal component effects (AMCEs) plotted with 95% confidence intervals. Panel A: Marginal means represent the average rating for decisions to penalize the account for each attribute level for two respondent subgroups: pro-freedom of expression and pro-mitigating misinformation. Dashed line represents the grand mean for rating (2.41). Panel B: AMCEs represent effects on rating to penalize the accounts for each attribute level, faceted by two respondent subgroups: Pro-freedom of expression and pro-mitigating misinformation. Dashed lines represent the null effect.

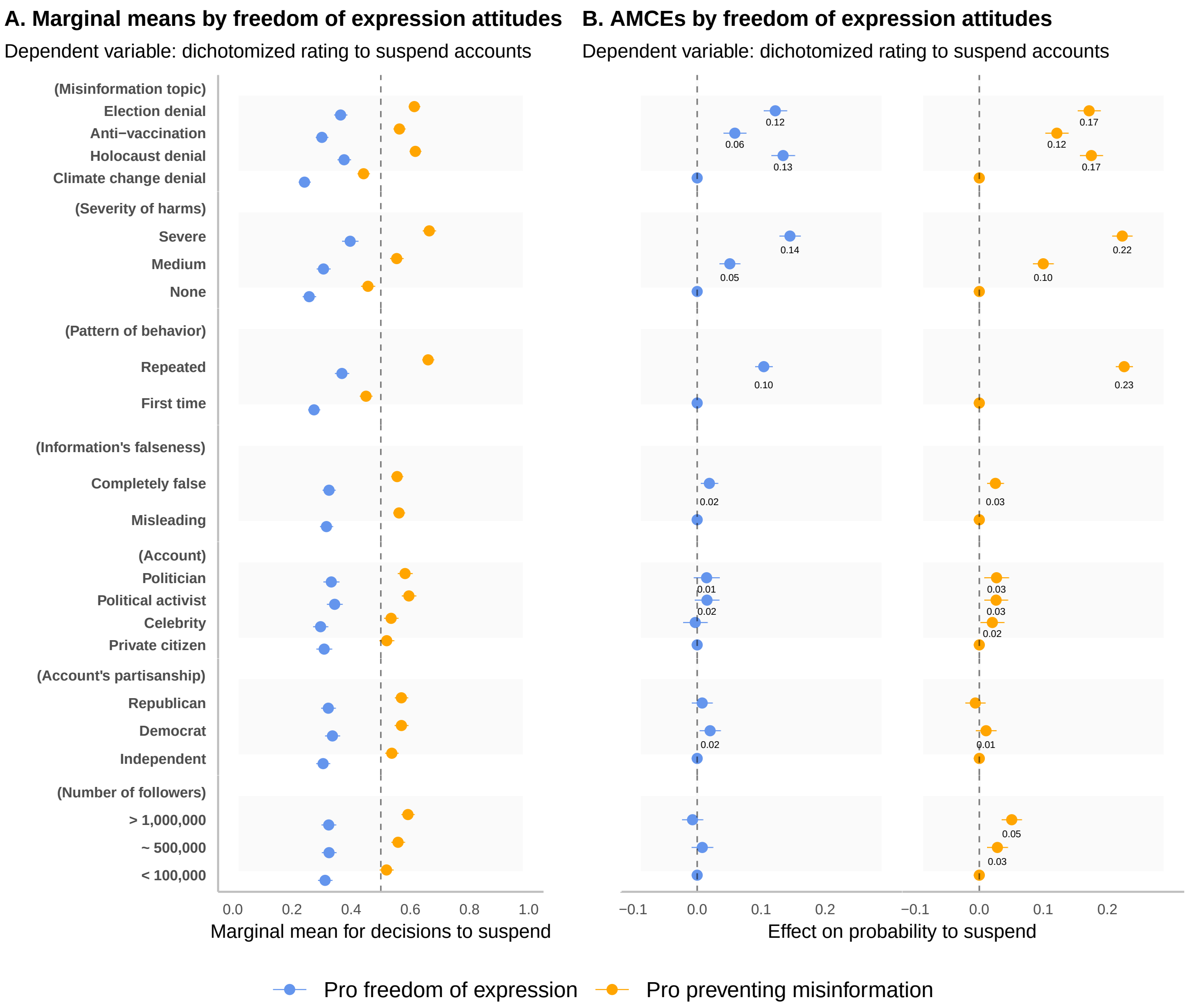

Fig. S9. *Respondent subgroup analyses: Dichotomized rating by respondents' attitudes toward free speech.* Marginal means point estimates and average marginal component effects (AMCEs) plotted with 95% confidence intervals. Panel A: Marginal means represent the average rating for decisions to penalize the account for each attribute level for two respondent subgroups: pro-freedom of expression and pro-mitigating misinformation. Dashed line represents the mean value for a binary decision (0.5). Panel B: AMCEs represent effects on rating to penalize the accounts for each attribute level, faceted by two respondent subgroups: Pro-freedom of expression and pro-mitigating misinformation. Dashed lines represent the null effect.

Appendix B: Descriptive and Summary Statistics on Survey Measures

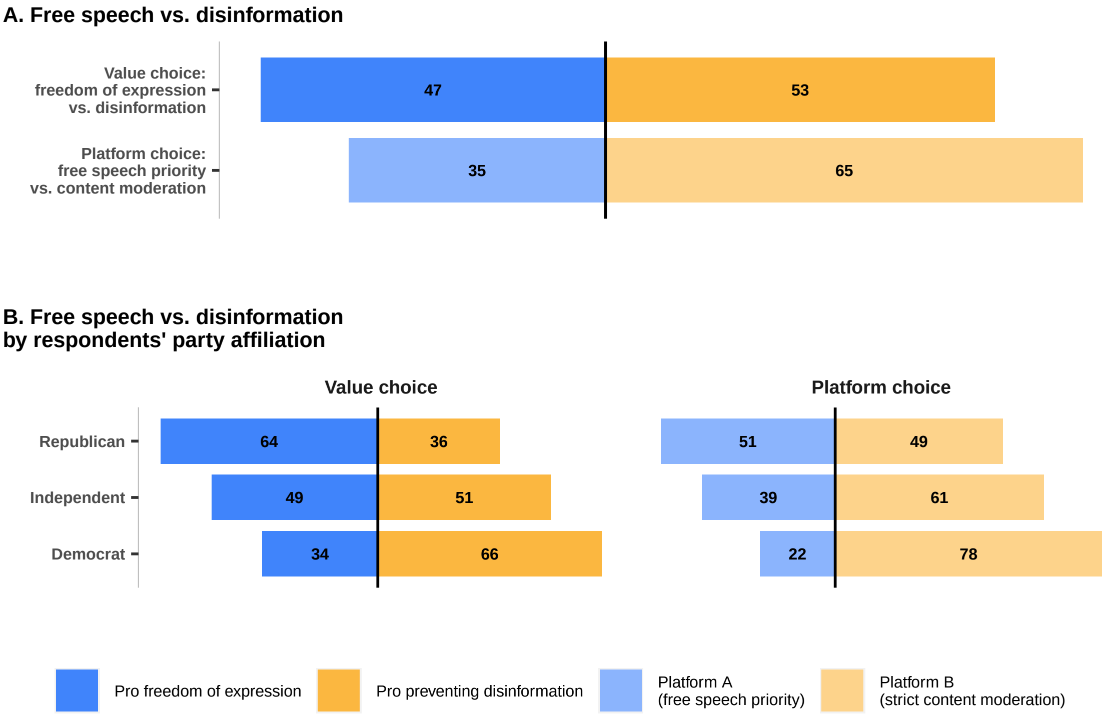

Fig. S10. *Preferences on freedom of expression for values and platforms.* Value choice: "If you absolutely have to choose between protecting freedom of expression and preventing disinformation from spreading, which is more important to you?" Platform choice: "Imagine you are considering joining one of two rival social media platforms. Platform A claims that it will always prioritize free speech and will never suspend an account or remove a post that incites violence, constitutes hate speech, or spreads false information. Platform B has a zero tolerance policy against false information, hate speech, and incitement to violence, and it will enforce strict content moderation rules for everyone. Which social media platform would you rather join?" Panel A: Proportion of responses for both questions for all participants. Panel B: Proportions by respondents' party affiliation.

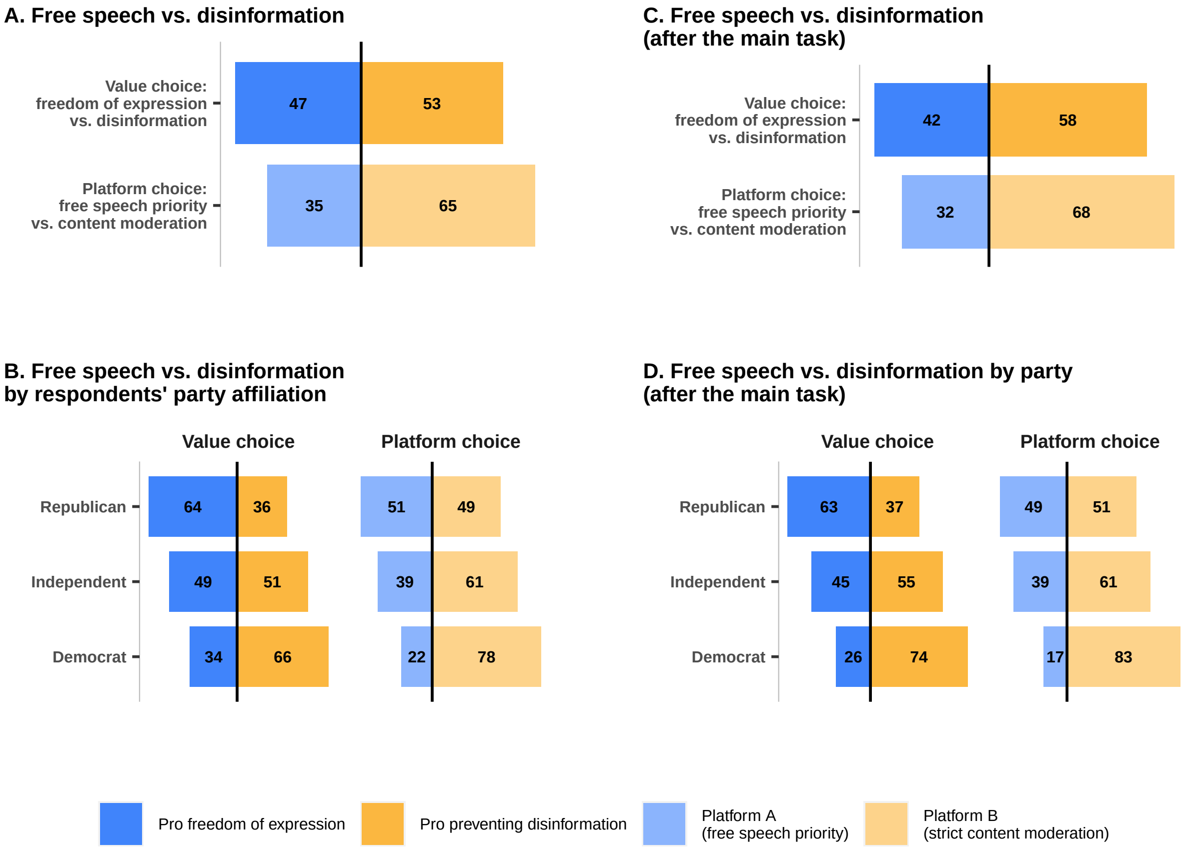

Fig. S11. *Freedom of expression versus mitigating misinformation: Before and after main task.* All numeric values represent percentages. Value choice: “If you absolutely have to choose between protecting freedom of expression and preventing disinformation from spreading, which is more important to you?”. Platform choice: “Imagine you are considering joining one of two rival social media platforms. Platform A claims that it will always prioritize free speech and will never suspend an account or remove a post that incites violence, constitutes hate speech, or spreads false information. Platform B has a zero tolerance policy against false information, hate speech, and incitement to violence, and it will enforce strict content moderation rules for everyone. Which social media platform would you rather join?” Panel A: Proportion of responses for both questions for all participants, before the main study task. Panel B: Proportions by party affiliation, before the main study task. Panel C: Proportion of responses for both questions for all participants, after the main study task. Panel D: Proportions by respondents’ party affiliation, after the main study task.

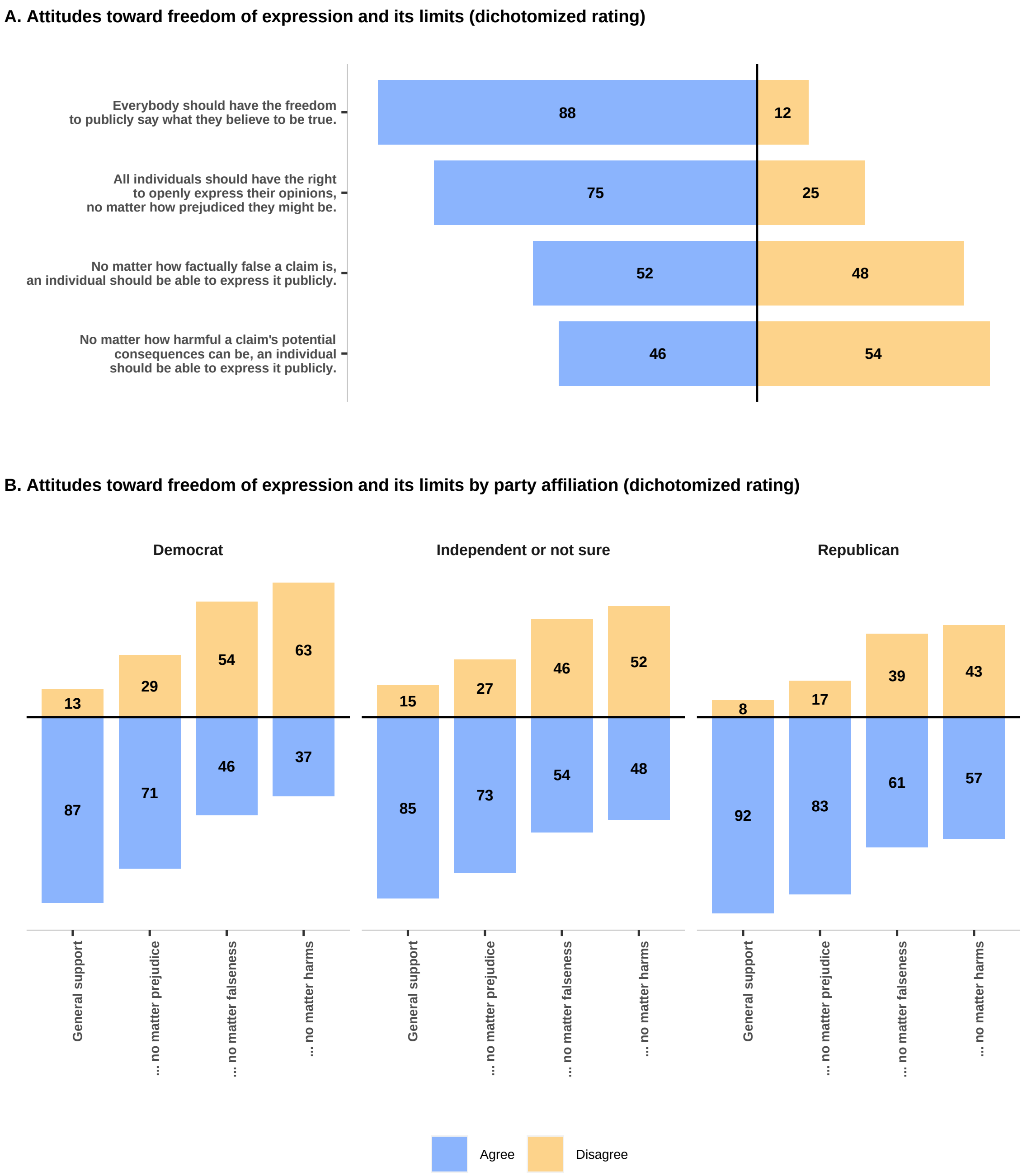

Fig. S12. *Freedom of expression and its limits.* All numeric values represent percentages. The four items addressed participants' general attitudes toward freedom of expression and its limits in cases of prejudice, falsehoods, and potential for harm on a 6-point Likert scale (Strongly disagree, Moderately disagree, Slightly disagree, Slightly agree, Moderately agree, Strongly agree). In this figure, these responses are grouped in two categories: Agree and Disagree. Panel A: Proportions of responses to four items querying general attitudes toward freedom of expression and its limits. Panel B: Proportions by respondents' party affiliation.

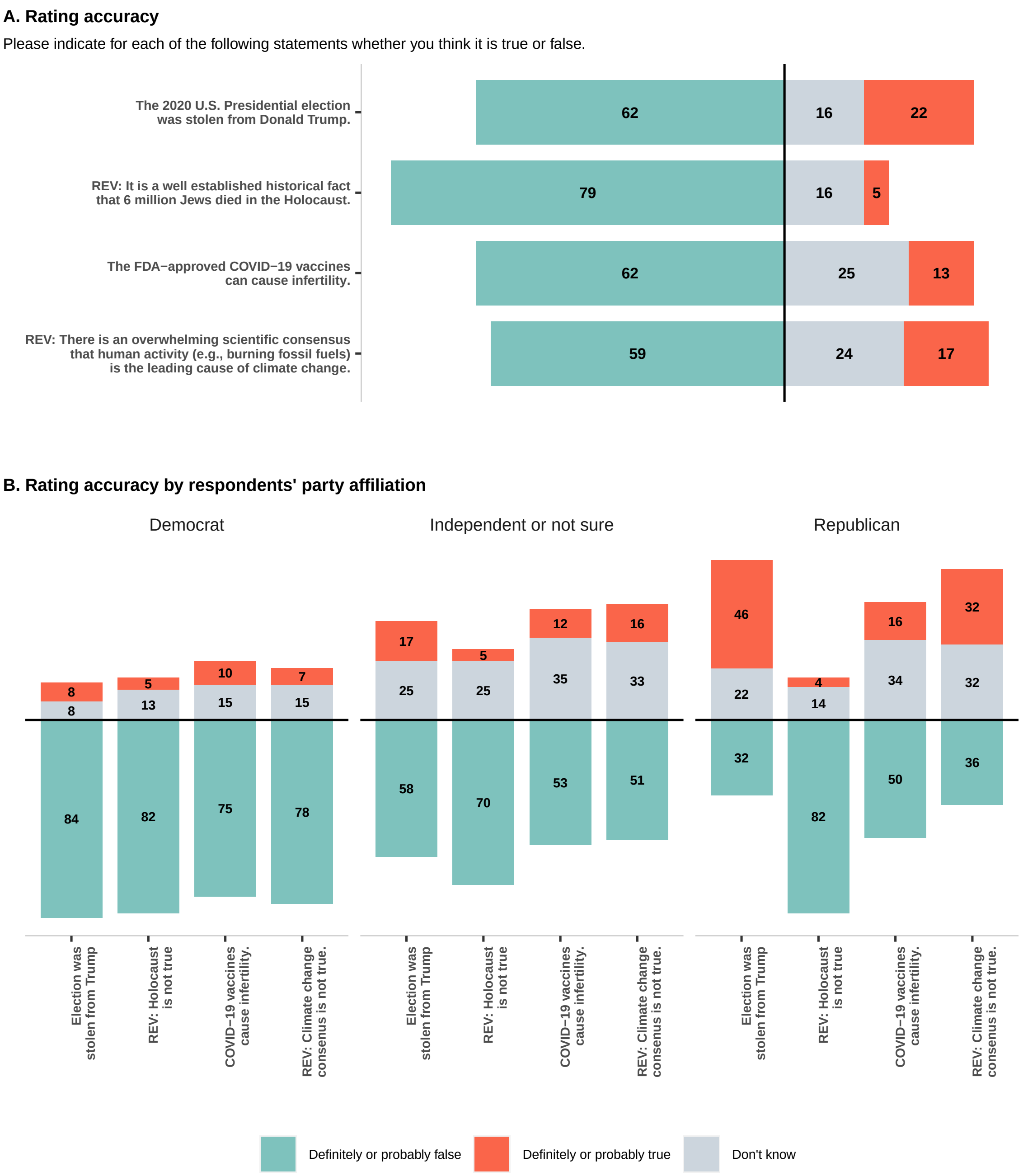

Fig. S13. *Accuracy ratings for misinformation statements.* All numeric values represent percentages. Panel A: Proportions of responses for rating accuracy of four claims on a 5-point Likert scale (definitely false, probably false, don't know, probably true, definitely true). Responses are grouped into three categories: Definitely or probably false: Do not know; Definitely or probably true. Responses for accurate statements are reverse coded (denoted by "REV" before the statement). Panel B: Proportions by respondents' party affiliation.

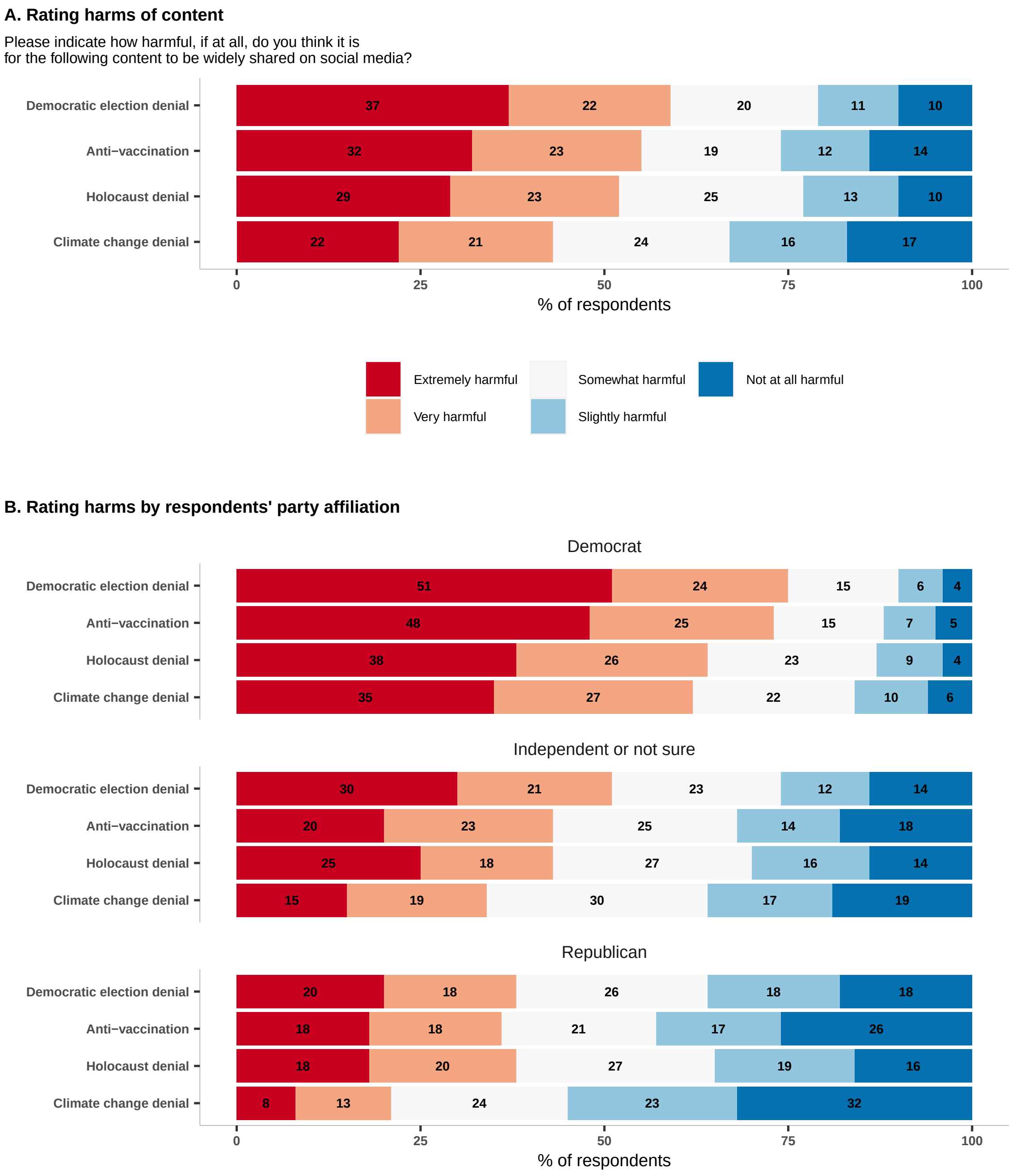

Fig. S14. *Content harm ratings for statements relevant to scenarios.* All numeric values represent percentages. Panel A: Proportions of responses for rating of perceived harm of the content featured in each scenario on a 5-point Likert scale (not at all harmful, slightly harmful, somewhat harmful, very harmful, extremely harmful). Panel B: Proportions by respondents' party affiliation.

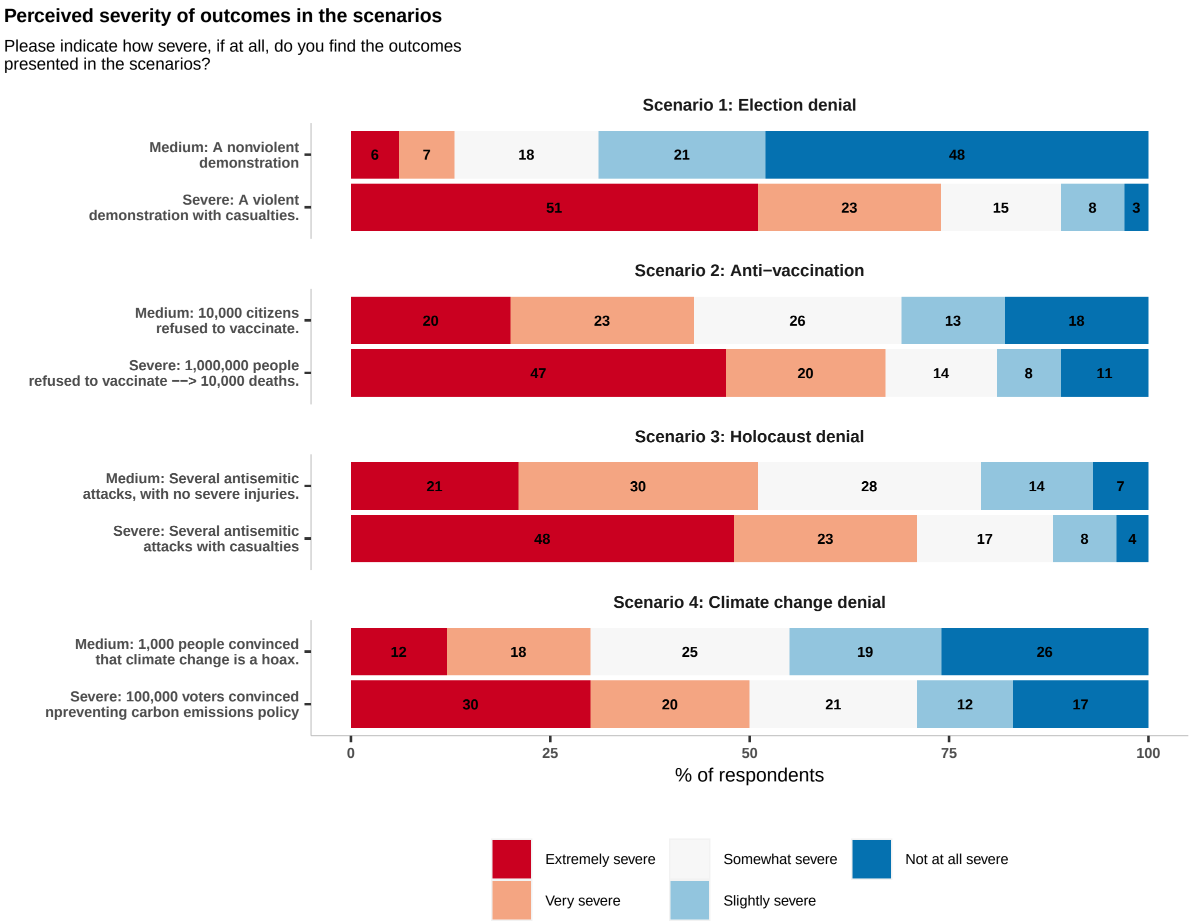

Fig. S15. *Perceived severity of outcomes in the scenarios.* All numeric values represent percentages. Proportions of responses for rating of perceived severity of the outcomes featured in the scenarios (on a 5-point Likert scale).

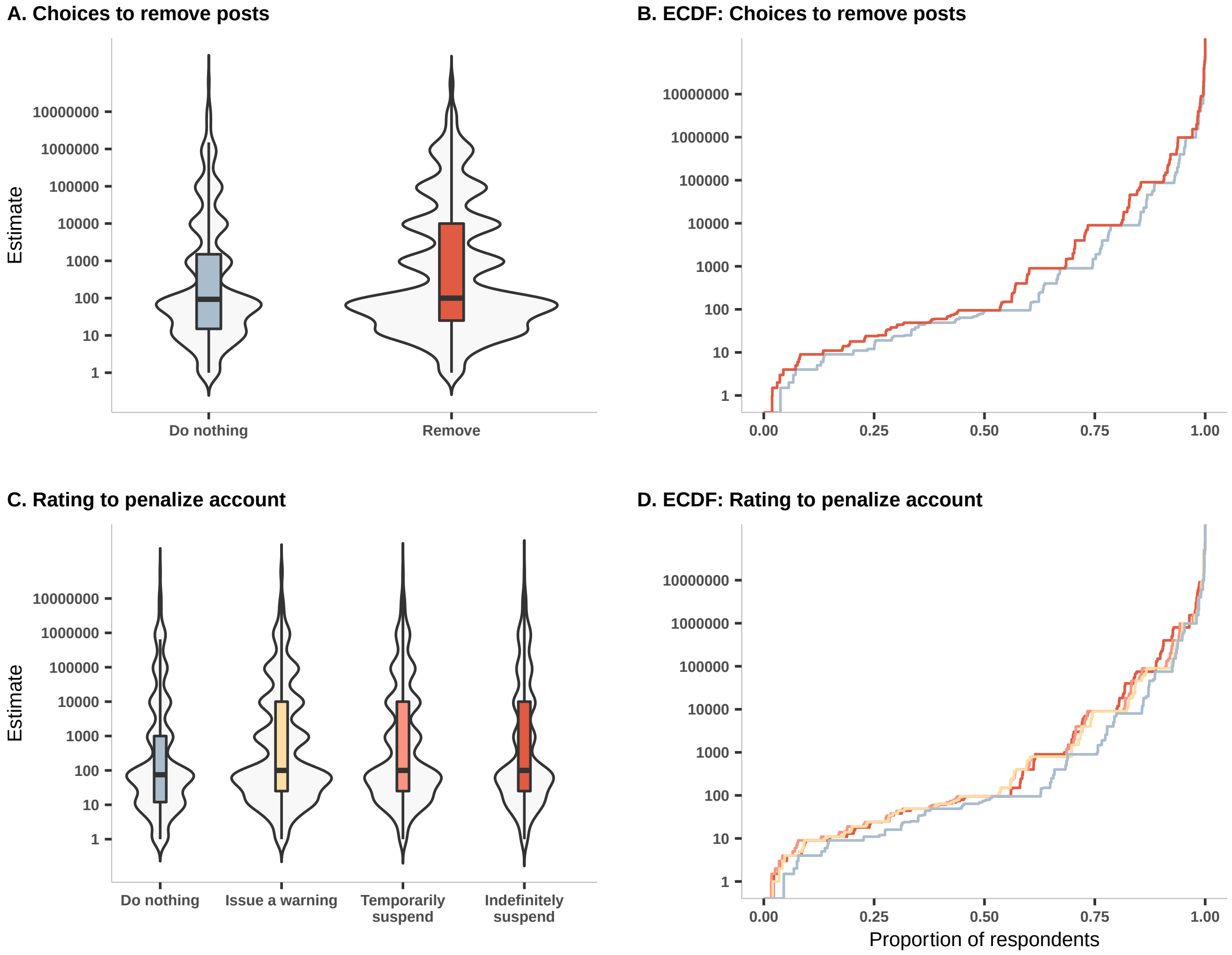

Fig. S16. *Summary statistics for relationship between estimates of number of disinformation accounts and outcome measures in the scenarios.* The estimation question was: "To the best of your knowledge, how many individuals are responsible for 65% of the anti-vaccination disinformation on Facebook and Twitter? Please indicate or estimate a number." Panel A: Boxplots and violin plots for the choice to remove posts. Panel C: Boxplots and violin plots for the choice to penalize account. Panel B: Empirical cumulative density function (ECDF) for the choice to remove posts. Panel D: Empirical cumulative density function (ECDF) for rating to penalize account.

Appendix C: Subset Analyses and Moderation Analyses by misinformation belief and partisanship

For the robustness analyses, we partitioned our conjoint data depending on whether a respondent's belief about the facts at hand was accurate or not. For instance, in the anti-vaccination scenario, which was displayed in 10,256 cases with different combinations of attributes, we excluded all conjoint data where a respondent indicated in the post-treatment question that the statement "The FDA-approved COVID-19 vaccines can cause infertility" was definitely or possibly true or if they did not know whether it was true or false (thus retaining 6,368 cases of this scenario). For the election denial scenario, we excluded profiles where respondents indicated in the post-treatment measure that the statement "The 2020 U.S. presidential election was stolen from Donald Trump" was definitely or possibly true or they did not know. In the Holocaust denial scenario, we excluded profiles where respondents indicated that the statement "It is a well established historical fact that 6 million Jews died in the Holocaust" was definitely or probably false or if they did not know. In the climate change denial scenario, we excluded profiles where respondents indicated that the statement "There is an overwhelming scientific consensus that human activity (e.g., burning fossil fuels) is the leading cause of climate change" was definitely or probably false or they did not know. From the total set of 40,845 cases we thus created a subset of 26,726 cases with accurate matching beliefs.

The main patterns in the subgroup differences persisted, including the finding that Republicans were less likely than independents and Democrats to take action against misinformation. However, as Figure S17 shows, more respondents with accurate beliefs than respondents with inaccurate or uncertain beliefs opted to remove false and misleading posts and to penalize accounts that spread them. In the subset (relative to the full dataset, see Figure S1), the election denial scenario and the anti-vaccination scenario overtook the Holocaust denial scenario in participants' likelihood to take action. However, these changes are difficult to interpret because the subset analysis excluded a participant's response if they endorsed inaccurate beliefs and the accuracy of beliefs systematically differed between the scenarios, the partisan groups, and their interaction.

Most Republicans with accurate beliefs were more likely than Republicans with inaccurate or uncertain beliefs to penalize online misinformation (but still less likely than independents and Democrats). For example, 64% of Republicans who rejected the statement "The 2020 U.S. Presidential election was stolen from Donald Trump" chose to remove the posts in the election denial scenario relative to 44% of the respondents who either endorsed the statement or chose the "do not know" response option—a difference of 20 percentage points (Figure S17). Even though differences in marginal means between respondents in the two subsets were large, they reveal heterogeneity of effects between these different populations only and do not allow for any causal claims.

To explore causal effects of misinformation beliefs and partisanship on respondents' content moderation preferences, we conducted a set of moderation analyses using the parallel estimation approach as defined in (1). The estimand of interest here was the average treatment moderation effect (ATME), which shows the effects of the nonrandomized moderators on randomized treatments (i.e., the conjoint attributes). For this analysis, we focused on two moderator variables: partisanship and accuracy of beliefs. Partisanship was measured in a pretreatment question and had three levels: Democrat, Independent, and Republican. Accuracy of beliefs was measured in a series of post-treatment questions in which respondents rated the accuracy of four scenario-specific statements (see Materials and Methods); it also had three levels: accurate beliefs (when respondents rated an inaccurate statement as false or an accurate statement as true), do not know, and inaccurate beliefs (when respondents rated an inaccurate statement as true or an accurate statement as false). The fact that this variable was post-treatment is a limitation in terms of the moderation analyses, as the ATME approach (1) assumes that nonrandomized moderators are measured pretreatment to avoid biased estimates. However, for the purposes of the current study it was more important to not prime our respondents with misinformation beliefs prior to the main study task. We also included three demographic measures (age, gender, and highest level of education) as control variables (i.e., covariates in the regression models). The moderation analysis focused on all conjoint attributes, except for the misinformation topic (scenario), because we could only conduct moderation analyses on the scenario level as the belief measures were scenario-specific. For each attribute, which acted as the randomly assigned treatment level in the parallel estimation process, we selected two levels (account: 0 = political, 1 = nonpolitical; account's partisanship: 0 = Democrat, 1 = Republican; number of followers: 0 = less than 1,000,000; 1 = more than 1,000,000; falseness of shared information: 0 = misleading, 1 = completely false; pattern of behavior: 0 = first time; 1 = repeated; severity of harm: 0 = none, 1 = severe).

The parallel estimation approach was implemented as follows: First, for each scenario, in each conjoint attribute, we subset the data by two levels (as specified above; e.g., in "severity of harms" by "none" and "severe"). Second, we estimated effects of the independent variables (partisanship, accuracy of beliefs, age, gender, education) on one of the two dependent variables (choice to remove posts and dichotomized rating of penalizing the account) in a linear regression for both subsets (e.g., "none" and "severe"). Third, for each estimated regression slope we computed the difference between the two respective estimates (which provided an estimate of the moderation effect) and its 95% confidence interval. See (1) for details on how to implement the ATME approach.

The aim was to investigate whether partisanship and accuracy of beliefs in misinformation relevant to our scenarios moderated the effects of conjoint attributes on respondents' content moderation decisions. Figures S18 and S19 show the results of these analyses for two dependent variables: choice to remove posts or do nothing, and rating to penalize the account, dichotomized to represent a choice between doing nothing/issuing a warning and temporarily/indefinitely suspending the account. The majority of estimates were nonsignificant and did not follow a clear pattern. One exception was the moderation by beliefs of the attributes "number of followers," "pattern of behavior," and "severity of harms" on decisions to suspend accounts (Figure S19A). Here, endorsing inaccurate claims ("The 2020 U.S. Presidential election was stolen from Donald Trump"; "The FDA-approved COVID-19 vaccines can cause infertility") and rejecting accurate claims ("There is an overwhelming scientific

consensus that human activity (e.g., burning fossil fuels) is the leading cause of climate change") made respondents on average less sensitive to the respective attribute levels, that is, when consequences of sharing misinformation were severe, when it was a repeated offense, and when an account had more than 1,000,000 followers. The same pattern did not hold, however, for the choice to remove posts (Figure S18A), with the exception of the "number of followers" attribute. Moreover, moderation effects of partisanship on effects of conjoint attribute "severity of harms" on decisions to remove, while largely insignificant, point in the opposite direction than beliefs (Figure S18B), making Republicans more sensitive to changes when consequences of sharing misinformation were severe. In decisions to suspend accounts, however, Republicans followed a similar pattern to that found for respondents who endorsed false claims for the attributes "pattern of behavior" and "number of followers."

Taken together, both robustness checks in the subset analyses and causal moderation analyses show that prior beliefs play a role in content moderation decisions but do not support the claim that these beliefs offer a viable explanation for the substantial differences in content moderation preferences between Republicans and Democrats that we observed.

A. Subset analysis: Choices by respondents with accurate and inaccurate or uncertain beliefs

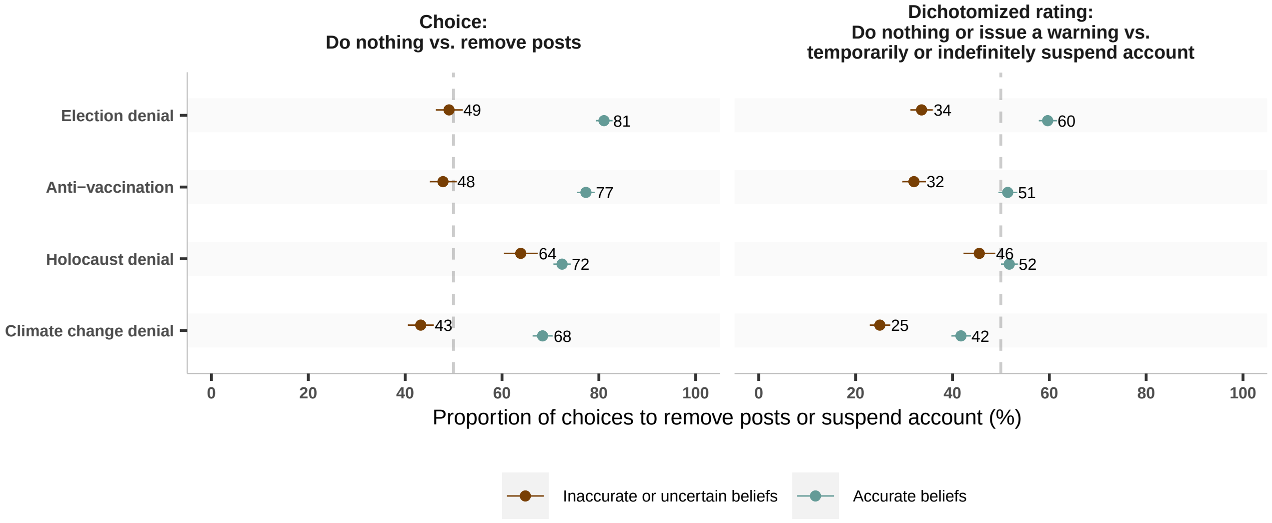

B. Subset analysis: Choices by respondents with accurate and inaccurate or uncertain beliefs by party

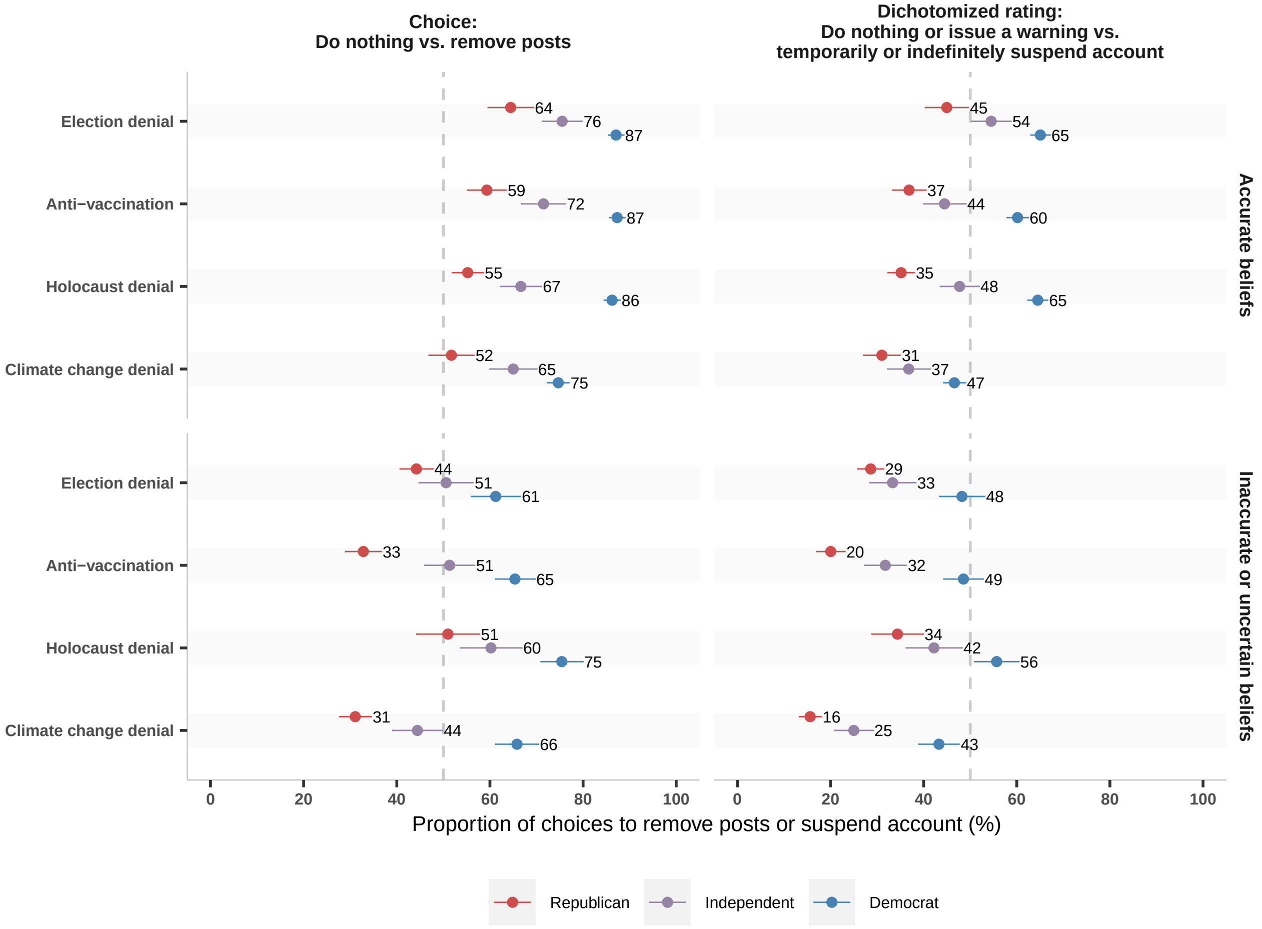

Fig. S17. *Subset of cases evaluated by respondents with accurate beliefs: Proportions.* All numeric values represent percentages. Panel A: Choices to remove the posts and suspend the accounts by misinformation topic and accuracy of beliefs. Panel B: Choices to remove posts and suspend accounts, by topic, party affiliation, and accuracy of beliefs. Total *N* of cases in the subset of respondents with accurate beliefs: 26,726 (evaluated by Democrats: 15,351; by independents: 4,769; by Republicans: 6,606). Total *N* of cases in the subset of respondents with inaccurate and uncertain beliefs: 14,119 (evaluated by Democrats: 3,987; by independents: 3,460; by Republicans: 6,672).

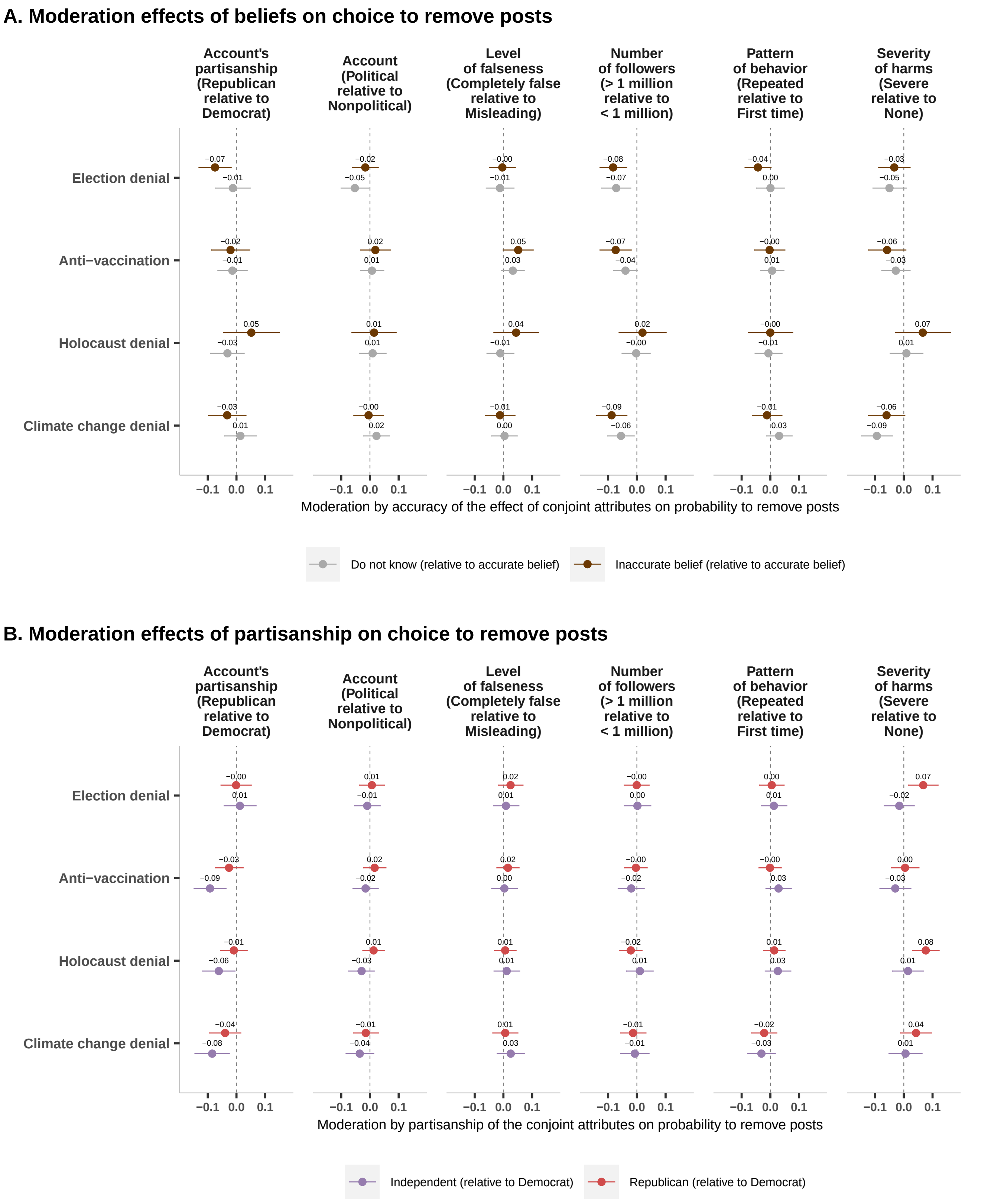

Fig. S18. *Moderation effects of misinformation beliefs and partisanship on decisions to remove posts.* All effects and 95% confidence intervals are estimated using parallel regression approach. Panel A: Moderation by accuracy of beliefs. Panel B: Moderation by partisanship.

A. Moderation effects of beliefs on dichotomized rating to suspend accounts

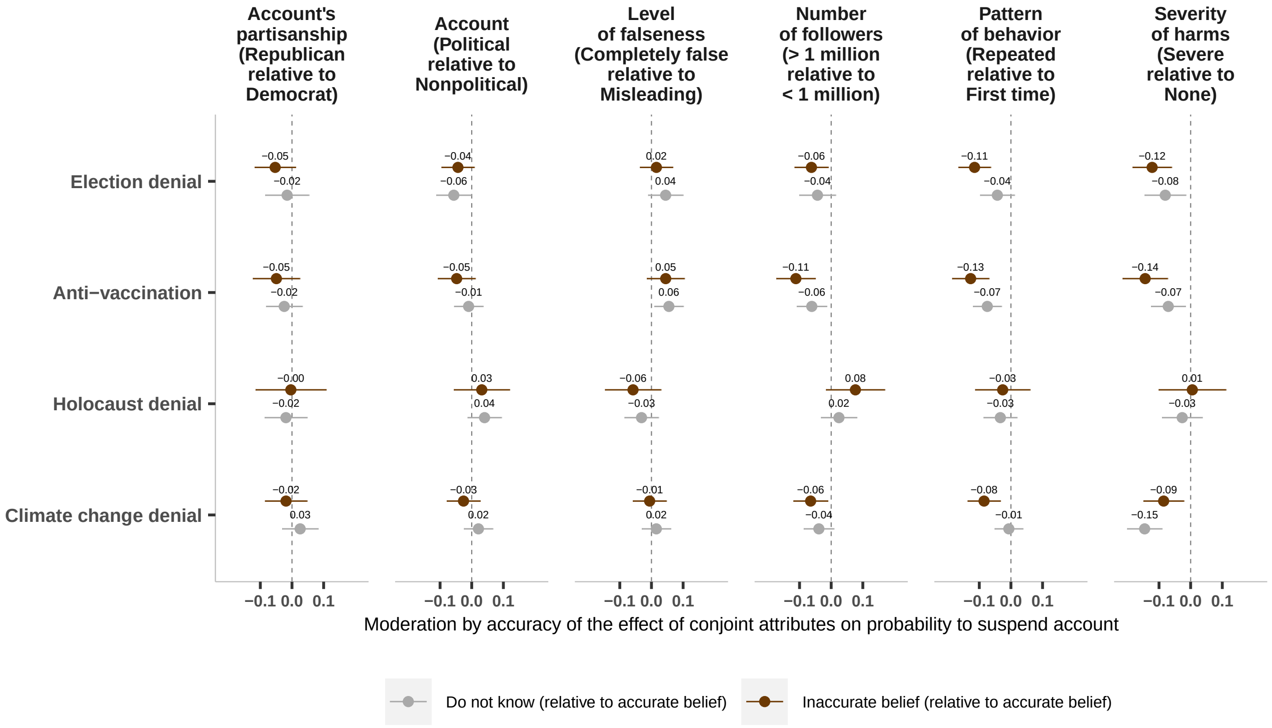

B. Moderation effects of partisanship on dichotomized rating to suspend accounts

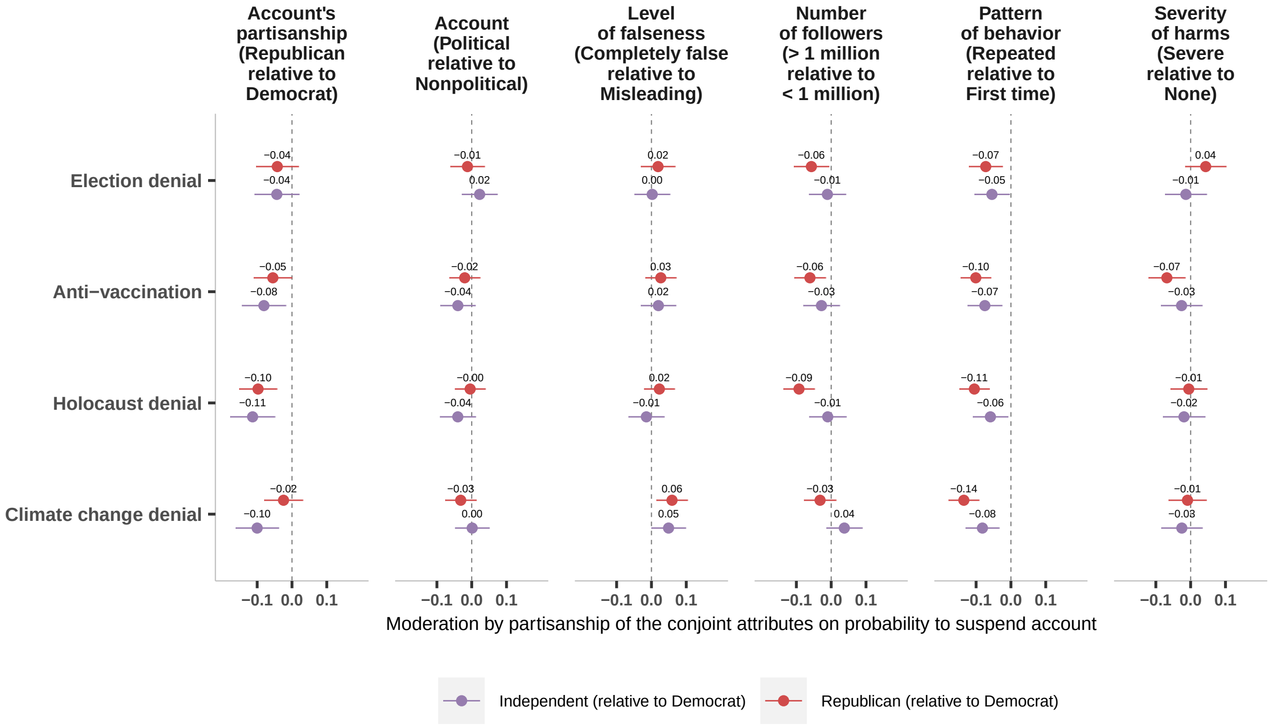

Fig. S19. *Moderation effects of misinformation beliefs and partisanship on decisions to suspend accounts.* All effects and 95% confidence intervals are estimated using parallel regression approach. Panel A: Moderation by accuracy of beliefs. Panel B: Moderation by partisanship.

Appendix D: Misinformation Policies

Table S10. Social Media Platforms' Misinformation Policies (last updated June 03, 2022)

Type of content	Platform	Policy	Strike system	Cases
COVID-19 misinformation	Google: Ads	Misrepresentation policy:<br>What is not allowed: "Content promoting harmful health claims, or content that relates to a current, major health crisis and contradicts authoritative scientific consensus. Examples (non-exhaustive): Anti-vaccine advocacy; denial of the existence of medical conditions such as AIDS or Covid-19; gay conversion therapy"(2)	"Violations of this policy will not lead to immediate account suspension without prior warning." A warning will be issued at least 7 days prior to suspension and appeal is possible." (2)	
	Google: YouTube	COVID-19 medical misinformation policy:<br>"YouTube doesn't allow content that spreads medical misinformation that contradicts local health authorities' (LHA) or the World Health Organization's (WHO) medical information about COVID-19." (3)<br><br>Vaccine misinformation policy:<br>"YouTube doesn't allow content that poses a serious risk of egregious harm by spreading medical misinformation about currently administered vaccines that are approved and confirmed to be safe and effective by local health authorities and by the World Health Organization (WHO)." (4)	Post deletion.<br>1st offense: No penalty, warning<br>2nd offense, 1 strike: 1 week ban on activity<br>2 strikes in 90 days: 2 week ban on posting<br>3 strikes in 90 days: Channel termination * (5)	"Since last year, we've removed over 130,000 videos for violating our COVID-19 vaccine policies."* (6)
	Meta: Ads	Misleading Claims Advertising Policy:<br>Prohibits ads that "make deceptive, false or unsubstantiated health claims, including claims that a product or service can provide 100% prevention or immunity, or is a cure for the virus." (7)	"Repeat offenders are subject to enforcement. If we see advertisers repeatedly violate our advertising policies, we may take action, including but not limited to, losing the ability to advertise via disablement of a single ad account, Ads Manager, Business Manager, Facebook Page or Instagram page." (7)	"Today, following consultations with leading health organizations, including the World Health Organization (WHO), we are expanding the list of false claims we will remove to include additional debunked claims about the coronavirus and vaccines. This includes claims such as: COVID-19 is man-made or manufactured; Vaccines are not effective at preventing the disease they are meant to protect against; It's safer to get the disease than to get the vaccine; Vaccines are toxic, dangerous or cause autism, The full list of claims is available here, and we already prohibit these claims in ads. These new policies will help us continue to take aggressive action against misinformation about COVID-19 and vaccines." (8)

Continued on next page

Table S10 – continued from previous page

Type of content	Platform	Policies	System of removal	Cases
	Meta: Facebook, Instagram	Restricted Goods and Services policy:<br>Prohibits posts that "indicate a sense of urgency or claims that prevention is guaranteed." (9)<br><br>Hate speech policy:<br>Prohibits posts that "state that people who share a protected characteristic such as race or religion have the virus, created the virus or are spreading the virus." (9)<br><br>Bullying and harassment policy:<br>Prohibits "claims that a private individual has COVID-19, unless that person has self-declared or information about their health status is publicly available." (9)<br><br>List of measures against COVID-19 misinformation:<br>"We remove COVID-19 related misinformation that could contribute to imminent physical harm." (10)	Posts violating community guidelines get deleted.<br><br>One strike: Warning and no further restrictions.<br>2 strikes: One-day restriction from creating content, such as posting, commenting, using Facebook Live or creating a Page.<br>3 strikes: 3-day restriction from creating content.<br>4 strikes: 7-day restriction from creating content.<br>5 or more strikes: 30-day restriction from creating content.<br><br>All strikes on Facebook or Instagram expire after one year.** (11)	"During the month of March, we displayed warnings on about 40 million posts related to COVID-19 on Facebook, based on around 4,000 articles by our independent fact-checking partners. When people saw those warning labels, 95% of the time they did not go on to view the original content. To date, we've also removed hundreds of thousands of pieces of misinformation that could lead to imminent physical harm. Examples of misinformation we've removed include harmful claims like drinking bleach cures the virus and theories like physical distancing is ineffective in preventing the disease from spreading." (8)
	Twitter	COVID-19 misleading information policy:<br>"Content that is demonstrably false or misleading and may lead to significant risk of harm (such as increased exposure to the virus, or adverse effects on public health systems) may not be shared on Twitter." (12)	Labeling (1 strike), Request for Tweet deletion (2 strikes).<br><br>1 strike: No account-level action<br>2 strikes: 12-hour account lock<br>3 strikes: 12-hour account lock<br>4 strikes: 7-day account lock<br>5 or more strikes: Permanent suspension *** (12)	"Since introducing these policies on March 18, we have removed more than 1,100 Tweets containing misleading and potentially harmful content from Twitter. Additionally, our automated systems have challenged more than 1.5 million accounts which were targeting discussions around COVID-19 with spammy or manipulative behaviors." (13)
	TikTok	Community guidelines - COVID-19:<br>"Misinformation is defined as content that is inaccurate or false. While we encourage our community to have respectful conversations about subjects that matter to them, we do not permit misinformation that causes harm to individuals, our community, or the larger public regardless of intent. Do not post, upload, stream, or share ... medical misinformation that can cause harm to an individual's physical health" (14)	1st violation: Warning; if the violation is under zero-tolerance policy, then automatic ban + may also block a device to help prevent future accounts from being created.<br><br>2nd violation: One or more of the following;<br>• Temporary ban (typically between 24 or 48 hours), depending on the severity of the violation and previous violations.<br>• Restrict the account to a view-only experience (typically between 72 hours or up to one week)<br>• Permanent ban<br><br>But: Accrued violations will expire from individuals' record over time **** (15)	"We removed 51,505 videos in the second half of 2020 for promoting COVID-19 misinformation. Of those videos, 86% were removed before they were reported to us, 87% were removed within 24 hours of being uploaded to TikTok, and 71% had zero views." (16)
	Spotify	Spotify Platform Rules:<br>"What to avoid: ... Content that promotes dangerous false or dangerous deceptive medical information that may cause offline harm or poses a direct threat to public health." (17)	"Breaking the rules may result in the violative content being removed from Spotify. Repeated or egregious violations may result in accounts being suspended and/or terminated." (17)	
	Pinterest	Community guidelines - Misinformation:<br>"We remove or limit distribution of false or misleading content that may harm Pinners' or the public's well-being, safety or trust, including: Medically unsupported health claims that risk public health and safety, including the promotion of false cures, anti-vaccination advice, or misinformation about public health or safety emergencies" (18)	"We make sure content meets our Community Guidelines through both automated processes and human review. Accounts may be suspended due to single or repeat violations of our Community Guidelines"***** (19)	

Continued on next page

Table S10 – continued from previous page

Type of content	Platform	Policies	System of removal	Cases
Democratic election denial and misinformation on the voting process	Google: Ads	Misrepresentation policy:<br>"The following is not allowed: Making claims that are demonstrably false and could significantly undermine participation or trust in an electoral or democratic process. Example (non-exhaustive): Information about public voting procedures, political candidate eligibility based on age or birthplace, election results, or census participation that contradicts official government records; incorrect claims that a public figure has died, or been involved in an accident." (2)	"Violations of this policy will not lead to immediate account suspension without prior warning." A warning will be issued at least 7 days prior to suspension and appeal is possible." (2)	"After review, and in light of concerns about the ongoing potential for violence, we removed new content uploaded to Donald J. Trump's channel for violating our policies. It now has its 1st strike & is temporarily prevented from uploading new content for a *minimum* of 7 days." (20)
	Google: YouTube	Community guidelines:<br>The content removed may include<br>"- Content that aims to mislead people about voting or the census processes, like telling viewers an incorrect voting date.<br>- Content that advances false claims related to the technical eligibility requirements for current political candidates and sitting elected officials to serve in office, such as false claims that a candidate is not eligible to hold office based on false information about citizenship status requirements to hold office in that country.<br>- Content that advances false claims that widespread fraud, errors, or glitches changed the outcome of any past U.S. presidential election." (21)	* (5)	
	Meta: Facebook, Instagram	Coordinating harm and publicising crime:<br>"In an effort to prevent and disrupt offline harm and copycat behaviour, we prohibit people from facilitating, organising, promoting or admitting to certain criminal or harmful activities targeted at people, businesses, property or animal. ... Do not post content that falls into the following categories: ... - Voter and/or census interference" (22)	** (11)<br><br>"When there is civil unrest, we may also restrict accounts by public figures for longer periods of time when they incite or praise ongoing violence. We'll determine the restriction period after assessing the severity of the violation, the account's history of past violations and the overall risk to public safety." (11)	"Given the gravity of the circumstances that led to Mr. Trump's suspension, we believe his actions constituted a severe violation of our rules which merit the highest penalty available under the new enforcement protocols. We are suspending his accounts for two years, effective from the date of the initial suspension on January 7 this year." (23)
	Twitter	Civic integrity policy:<br>"You may not use Twitter's services for the purpose of manipulating or interfering in elections or other civic processes. This includes posting or sharing content that may suppress participation or mislead people about when, where, or how to participate in a civic process. In addition, we may label and reduce the visibility of Tweets containing false or misleading information about civic processes in order to provide additional context." (24)	*** (24)	"After close review of recent Tweets from the @realDonaldTrump account and the context around them — specifically how they are being received and interpreted on and off Twitter — we have permanently suspended the account. ... President Trump's statement that he will not be attending the Inauguration is being received by a number of his supporters as further confirmation that the election was not legitimate and is seen as him disavowing his previous claim made via two Tweets (1, 2) by his Deputy Chief of Staff, Dan Scavino, that there would be an "orderly transition" on January 20th." (25)
	TikTok	Community guidelines - Election integrity:<br>"Misinformation is defined as content that is inaccurate or false. While we encourage our community to have respectful conversations about subjects that matter to them, we do not permit misinformation that causes harm to individuals, our community, or the larger public regardless of intent. Do not post, upload, stream, or share: ... - Content that misleads community members about elections or other civic processes ... - Misinformation related to emergencies that induces panic." (26)	**** (15)	"In the second half of 2020, 347,225 videos were removed in the US for election misinformation, disinformation, or manipulated media. We worked with fact checkers at PolitiFact, Lead Stories, and SciVerify to assess the accuracy of content and limit distribution of unsubstantiated content. As a result, 441,028 videos were not eligible for recommendation into anyone's For You feed. We further removed 1,750,000 accounts that were used for automation during the timeframe of the US elections." (16)

Continued on next page

Table S10 – continued from previous page

Type of content	Platform	Policies	System of removal	Cases
	Spotify	Spotify Platform Rules:<br>"Content that attempts to manipulate or interfere with election-related processes includes, but may not be limited to:<br>- misrepresentation of procedures in a civic process that could discourage or prevent participation<br>- misleading content promoted to intimidate or suppress voters from participating in an election" (17)	"Breaking the rules may result in the violative content being removed from Spotify. Repeated or egregious violations may result in accounts being suspended and/or terminated." (17)	
	Pinterest	Community gudelines - Civic participation misinformation:<br>"We remove or limit distribution of false or misleading content that may harm Pinners' or the public's well-being, safety or trust, including: False or misleading content that impedes an election's integrity or an individual's or group's civic participation ...<br>- about who can vote or participate in the census and what information must be provided to participate" (18)	***** (19)	
Holocaust denial	Google: YouTube	Hate speech policy:<br>"Don't post content on YouTube if the purpose of that content is to do one or more of the following: ... - Deny that a well-documented, violent event took place." (27)	* (5)<br><br>And additionally: "If we think your content comes close to hate speech, we may limit YouTube features available for that content" (27) with no: comments, suggested videos, likes.	In an interview with the NPR national security correspondent Hannah Allam: "Well, there was no waiting around. This policy kicked in immediately. YouTube videos with extremist content started vanishing - videos that promoted white supremacy, neo-Nazi videos. Some civil rights groups and people who've been targeted for harassment online say it's a step in the right direction, although they also have concerns that it doesn't go far enough or it's impossible to enforce. And on the flipside, there are people who say it goes too far." (28)
	Meta: Facebook, Instagram	Hate speech policy:<br>Do not post "Designated dehumanising comparisons, generalisations or behavioural statements (in written or visual form) that include: ... - Denying or distorting information about the Holocaust." (29)	** (22)	
	Twitter	Abusive behavior policy:<br>"We prohibit content that denies that mass murder or other mass casualty events took place, where we can verify that the event occured, and when the content is shared with abusive intent. This may include references to such an event as a "hoax" or claims that victims or survivors are fake or "actors." It includes, but is not limited to, events like the Holocaust, school shootings, terrorist attacks, and natural disasters." (30)	"When determining the penalty for violating this policy, we consider a number of factors including, but not limited to the severity of the violation and an individual's previous record of rule violations. The following is a list of potential enforcement options for content that violates this policy:<br>- Downranking Tweets in replies, except when the user follows the Tweet author.<br>- Making Tweets ineligible for amplification in Top search results and/or on timelines for users who don't follow the Tweet author.<br>- Excluding Tweets and/or accounts in email or in-product recommendations.<br>- Requiring Tweet removal.<br>- Suspending accounts."<br>(30)	
	TikTok	Community Guidelines - Hateful behavior:<br>"Do not post, upload, stream, or share: ... Content that denies well-documented and violent events have taken place affecting groups with protected attributes." (31)	**** (15)	
	Spotify	No policy yet.		

Continued on next page

Table S10 – continued from previous page

Type of content	Platform	Policies	System of removal	Cases
	Pinterest	Community gudelines - Hateful activities: "We limit the distribution of or remove such content and accounts, including: Hate-based conspiracy theories and misinformation, like Holocaust denial" (18)	***** (19)	
Climate change denial	Google: Ads	Misrepresentation policy: "We want users to trust the ads on our platform, so we strive to ensure ads are clear and honest, and provide the information that users need to make informed decisions. We don't allow ads or destinations that deceive users by excluding relevant product information or providing misleading information about products, services, or businesses [e.g.,] - Making claims that contradict authoritative, scientific consensus on climate change" (2)	- Ad or extension disapproval until the issue is resolved<br>- Account suspension with (notification will be sent at least 7 days prior to suspension action) or without warning (if and only if egregious violation of the Google Ads policies happens)<br>- Remarketing list disabling<br>- Compliance review of the profile (32)	
	Google: YouTube	No policy yet.		
	Meta: Facebook, Instagram	No policy yet.		"We have a responsibility to tackle climate misinformation on our services, which is why we partner with more than 80 independent fact-checking organizations globally to review and rate content, including content about climate change. When they rate content as false, we reduce its distribution so fewer people see it and we show a warning label with more context. And we apply penalties to people who repeatedly share false information." (33)
	Twitter	No policy yet.		
	TikTok	No policy yet.		
	Spotify	No policy yet.		
	Pinterest	Community gudelines - Climate misinformation: "We remove or limit distribution of false or misleading content that may harm Pinners' or the public's well-being, safety or trust, including:<br>- Content that denies the existence or impacts of climate change, the human influence on climate change, or that climate change is backed by scientific consensus.<br>- False or misleading content about climate change solutions that contradict well-established scientific consensus.<br>- Content that misrepresents scientific data, including by omission or cherry-picking, in order to erode trust in climate science and experts." (18)	***** (19)	

References

1. K Bansak, Estimating causal moderation effects with randomized treatments and non-randomized moderators. *J. Royal Stat. Soc. Ser. A* **184**, 65–86 (2021).
2. Google, Misrepresentation, https://support.google.com/adspolicy/answer/6020955?hl=en (n.d.) Accessed: 2021-12-27.
3. YouTube, COVID-19 medical misinformation policy, https://support.google.com/youtube/answer/9891785?hl=en (n.d.) Accessed: 2021-12-26.
4. Google, Vaccine misinformation policy, Retrieved December 26, 2021, from https://support.google.com/youtube/answer/11161123 (n.d.).
5. Google, Community Guidelines strike basics, Retrieved December 26, 2021, from https://support.google.com/youtube/answer/2802032 (n.d.).
6. The YouTube Team, Managing harmful vaccine content on YouTube, https://blog.youtube/news-and-events/managing-harmful-vaccine-content-youtube/ (2021).
7. Meta, Advertising policies related to coronavirus (COVID-19), Retrieved January 4, 2022, from https://www.facebook.com/business/help/1123969894625935 (n.d.).
8. G Rosen, An update on our work to keep people informed and limit misinformation about COVID-19, https://about.fb.com/news/2020/04/covid-19-misinfo-update/#removing-more-false-claims (2020) Accessed: 2022-01-04.
9. Instagram, COVID-19 and vaccine policy updates and protections, https://help.instagram.com/697825587576762 (n.d.) Accessed: 2021-12-27.
10. N Clegg, Combating COVID-19 misinformation across our apps, https://about.fb.com/news/2020/03/combating-covid-19-misinformation/ (2020).
11. Meta, Counting strikes, Retrieved December 26, 2021, from https://transparency.fb.com/en-gb/enforcement/taking-action/counting-strikes/ (n.d.).
12. Twitter, COVID-19 misleading information policy, https://help.twitter.com/en/rules-and-policies/medical-misinformation-policy (n.d.) Accessed: 2021-12-26.
13. Twitter, Coronavirus: Staying safe and informed on twitter, Retrieved December 27, 2021, from https://blog.twitter.com/en_us/topics/company/2020/covid-19 (2020).
14. TikTok, COVID-19, Retrieved December 26, 2021, from https://www.tiktok.com/safety/en-us/covid-19/ (n.d.).
15. TikTok, Content violations and bans, Retrieved December 26, 2021, from https://support.tiktok.com/en/safety-hc/account-and-user-safety/content-violations-and-bans (n.d.).
16. TikTok, Community guidelines enforcement report, https://www.tiktok.com/safety/resources/transparency-report-2020-2?lang=en (2021).
17. Spotify, Spotify platform rules, https://newsroom.spotify.com/2022-01-30/spotify-platform-rules/ (2022).
18. Pinterest, Community guidelines, https://policy.pinterest.com/en/community-guidelines (n.d.) Accessed: 2022-04-26.
19. Pinterest, Account suspension, Retrieved April 24, 2022, from https://help.pinterest.com/en/article/account-suspension (n.d.).
20. YouTube Insider, After review, and in light of concerns about the ongoing potential for violence, we removed new content uploaded to Donald J. Trump's channel for violating our policies. It now has its 1st strike & is temporarily prevented from uploading new content for a *minimum* of 7 days., https://twitter.com/YouTubeInsider/status/1349205688694812672?s=20&t=pMA3f60oCs6NI5ALZuI-Zw (2021) Tweet.
21. YouTube, How does YouTube support civic engagement and stay secure, impartial, and fair during elections?, Retrieved February 11, 2022, from https://www.youtube.com/intl/en_us/howyoutubeworks/our-commitments/supporting-political-integrity/ (n.d.).
22. Meta, Coordinating harm and promoting crime, Retrieved December 26, 2021, from https://transparency.fb.com/en-gb/policies/community-standards/coordinating-harm-publicizing-crime/ (n.d.).
23. N Clegg, In response to Oversight Board, Trump suspended for two years; will only be reinstated if conditions permit, https://about.fb.com/news/2021/06/facebook-response-to-oversight-board-recommendations-trump/ (2021).
24. Twitter, Civic integrity policy, Retrieved December 26, 2021, from https://help.twitter.com/en/rules-and-policies/election-integrity-policy (n.d.).
25. Twitter, Permanent suspension of @realDonaldTrump, https://blog.twitter.com/en_us/topics/company/2020/suspension (2021).
26. TikTok, Election integrity, Retrieved December 26, 2021, from https://www.tiktok.com/safety/en-us/election-integrity/ (2020).
27. Google, Hate speech policy, Retrieved December 27, 2021, from https://support.google.com/youtube/answer/2801939?hl=en (n.d.).
28. L Garcia-Navarro, YouTube removes white supremacist content, https://www.npr.org/2019/06/09/731044416/youtube-removes-white-supremacist-content?t=1643586899974 (2019).
29. Meta, Hate speech, Retrieved December 27, 2021, from https://transparency.fb.com/en-gb/policies/community-standards/hate-speech/ (n.d.).
30. Twitter, Abusive behavior, Retrieved January 4, 2022, from https://help.twitter.com/en/rules-and-policies/abusive-behavior (n.d.).
31. TikTok, Community guidelines, Retrieved December 27, 2021, from https://www.tiktok.com/community-guidelines?lang=en (2020).
32. Google, What happens if you violate our policies, Retrieved December 27, 2021, from https://support.google.com/adspolicy/answer/7187501?hl=en&ref_topic=1308266 (n.d.).

33. N Clegg, Our commitment to combating climate change, https://about.fb.com/news/2021/11/our-commitment-to-combating-climate-change/ (2021).
